# Probing and harnessing photonic Fermi arc surface states using light-matter interactions

**DOI:** 10.1126/sciadv.adf8257

**Published:** 2023-05-31

**Authors:** Iñaki García-Elcano, Jaime Merino, Jorge Bravo-Abad, Alejandro González-Tudela

**Affiliations:** ^1^ Departamento de Física Teórica de la Materia Condensada and Condensed Matter Physics Center (IFIMAC), Universidad Autónoma de Madrid, E-28049 Madrid, Spain.; ^2^ Instituto de Física Fundamental (IFF), CSIC, Calle Serrano 113b, Madrid 28006, Spain.

## Abstract

Fermi arcs, i.e., surface states connecting topologically distinct Weyl points, represent a paradigmatic manifestation of the topological aspects of Weyl physics. We investigate a light-matter interface based on the photonic counterpart of these states and prove that it can lead to phenomena with no analog in other setups. First, we show how to image the Fermi arcs by studying the spontaneous decay of one or many emitters coupled to the system’s border. Second, we demonstrate that, exploiting the negative refraction of these modes, the Fermi arc surface states can act as a robust quantum link, enabling, e.g., the occurrence of perfect quantum state transfer between the considered emitters or the formation of highly entangled states. In addition to their fundamental interest, our findings evidence the potential offered by the photonic Fermi arc light-matter interfaces for the design of more robust quantum technologies.

## Teaser

The great potential of a light-matter interface based on the surface states of a Weyl photonic system is studied.

## INTRODUCTION

The introduction of topology to explain the observation of quantized electron transport (*1*) has led to a revolution in Physics, permeating in fields beyond condensed-matter, such as photonics (*2*) or acoustics (*3*). On the fundamental side, it has brought the discovery that certain phases of matter can only be characterized by global order parameters (*4*), escaping thus to the Ginzburg-Landau paradigm. From a more applied standpoint, such topological phases are accompanied by the appearance of topological boundary states. Owing to their topological origin, these boundary states are immune to disorder and thus can be used to engineer robust devices. Initially, the field focused on topological phases and their boundary states in one and two dimensions, such as two-dimensional (2D) Chern (ℤ_2_) insulators and their chiral (helical) (*5*) edge modes (*6*). However, the observation in 2008 of the first 3D topological insulator (*7*) and in 2015 of Weyl semimetals in electronic (*8*, *9*) and photonic (*10*) setups has driven the attention to the 3D case (*11*, *12*).

Weyl systems, in particular, stand as one of the most paradigmatic examples of a 3D topological phase. They are characterized by the presence of several single-point linear degeneracies in their bulk spectrum, known as Weyl points, which have associated a quantized Berry curvature. Such quantization triggers the appearance of topological surface states with an energy dispersion connecting two topologically inequivalent Weyl points: the Fermi arcs (*13*). These unconventional surface modes are responsible for exotic phenomena in electronic systems such as bulk-mediated quantum oscillations (*14*), as well as unusual classical wave propagation in bosonic settings such as photonics or acoustics (*15*–*19*). However, an important practical difference between the two scenarios is that whereas the electronic ground state fills up until the Fermi level and thus naturally probes Fermi arc energies, bosonic excitations accumulate in the lowest energy state because of their statistics. Thus, the phenomena and detection schemes introduced in the electronic context cannot be, in general, directly extrapolated to the bosonic ones.

In photonics, this limitation, which is commonplace for all topological models, is motivating interfacing such structures with emitters (*20*–*23*). The reason is that emitters can probe the photonic system at topologically nontrivial frequency regions, making them active. As an added value, emitters are strongly interacting systems that can induce photonic interactions through light-matter couplings. These recent experimental developments are driving many theoretical studies, which, so far, have focused on understanding the photon-mediated interactions when emitters couple to topological bulk modes (*22*, *24*–*31*). Recent studies with 1D (*32*) and 2D topological photonic systems (*33*) have discovered how the interaction of emitters with the topological boundary modes can also lead to exciting regimes in cavity and waveguide quantum electrodynamics (QED). The interaction of topological surface states with emitters has not yet been studied, and thus, their potential applications still remain an open question.

In this work, we undertake this endeavor by characterizing a Fermi arc light-matter interface, consisting of a set of emitters coupled to the edges of a Weyl system, and find several unexpected phenomena. First, we demonstrate that one can directly image the Fermi arcs by monitoring the free-space spontaneous emission of the emitters. Second, we prove that the surface modes can act as a robust quantum link connecting the emitters in two different ways. In the infinite size limit, we show how to engineer the negative refraction (NR) occurring at the hinges of the system to obtain a perfect, dissipative, chiral channel (*34*), and characterize its performance by studying the spontaneous formation of entanglement. We demonstrate that the studied channel can reach the maximum entangling capacity of a perfect chiral waveguide (*35*), which opens up its use for quantum state transfer (*36*) or to obtain driven-dissipative maximally entangled states (*37*–*39*). For small systems, meaning that revivals of the emitters occur, the multiple NRs taking place at the corners of the structure facilitate the formation of a closed photonic path that induces an effective cavity mode. Such cavity leads to perfect coherent exchanges between the emitters, which can be used to maximally entangle them in a time-dependent fashion.

## RESULTS

### Fermi arc light-matter interfaces

In this section, we provide all the details about the Fermi arc light-matter interface that we will consider in this manuscript. First, we briefly describe the light-matter Hamiltonian that is used. Then, we make an extensive description of the considered photonic Weyl environment, explaining its bulk and boundary properties.

#### Light-matter coupling scheme

**Fig. 1.**
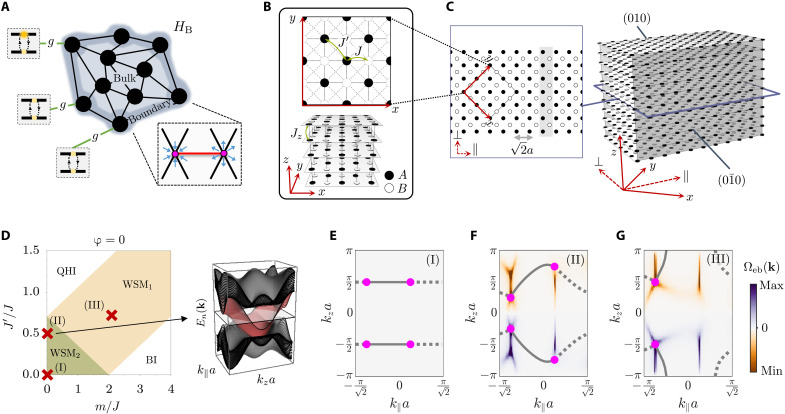
Photonic Weyl environment realized in a 3D lattice of localized bosonic modes. (**A**) Schematic view of the investigated system. (**B**) Hopping amplitudes associated with the intralayer (top) and interlayer (bottom) interactions in the discrete lattice model. Black and white shallow cylinders represent the localized bosonic modes belonging to the *A* and *B* sublattices, respectively. (**C**) Slab model for the considered cut. Left: The *z* = 0 plane of the photonic lattice, where the shadowed gray area represents the unit cell associated with the cut system. Right: 3D view of the slab. As seen, we choose both the $\left(0\bar{1}0\right)$ and (010) facets to be composed by sites belonging to the *A* sublattice. (**D**) Phase diagram of the Weyl photonic environment setting ϕ = 0 (see the definition in Eq. 5), as a function of the on-site energy offset between sublattices *m* and the next-nearest-neighbor hoppings *J*′. The inset shows the band structure corresponding to the (II) configuration, where the red surface represents the edge band. (**E** to **G**) Fermi arcs associated with three different configurations marked by red crosses in the phase diagram displayed in (D). Solid and dotted lines differentiate between states associated with the $\left(0\bar{1}0\right)$ and the (010) facets, respectively. Magenta points denote the projection of the Weyl singularities over the surface Brillouin zone. The color map indicates the Berry curvature calculated for the edge band Ω_eb_(**k**), in each of the considered cases. For the calculation, a slab of width $w/a=16\sqrt{2}$ (i.e., 33 non-equivalent sites per unit cell) is used. Therefore, the edge band is identified as the *n* = 17 band. Hot lines along which the surface Berry curvature displays a nontrivial behavior are present in (F) and (G).

A schematic overview of the system under study is depicted in Fig. 1A. We consider one or more emitters coupled to the boundary of a photonic Weyl environment, which we model as a discrete photonic lattice. The interplay among the modes spanning such topological reservoir is captured by a tight-binding Hamiltonian of the form (taking ℏ = 1 hereafter)

$$H_{B}=\sum_{{rr}^{\prime }}J_{{rr}^{\prime }}a_{r}^{†}a_{r^{\prime }}$$ where $a_{r}(a_{r}^{†})$ annihilates (creates) a bosonic excitation at position **r** and *J*_**rr**′_ is, in the most general case, a complex hopping matrix element. The band structure resulting from the diagonalization of *H*_B_ harbours an even number of Weyl points. These are point-like degeneracies in reciprocal space around which, assuming that we limit ourselves to the study of the so-called type I Weyl semimetals with no tilting velocity terms (*12*, *40*), the dispersion can be expanded as follows

$$E_{\pm }(k∼k_{W}^{i})\approx \omega_{W}\pm \sqrt{\sum_{\alpha \alpha^{\prime }=x,y,z}{\bar{M}}_{\alpha \alpha^{\prime }}^{i}\;q_{\alpha }^{i}q_{\alpha^{\prime }}^{i}}$$ where we define ω*_W_* as the Weyl frequency, $q^{i}=k-k_{W}^{i}$ denotes the distance between an arbitrary point in the Brillouin zone **k** and the position of the *i*-th Weyl node $k_{W}^{i}$, and ${\bar{M}}^{i}$ is a positive definite matrix.

For the emitters, we use the simplest description, that is, considering them as two-level systems with resonant frequency ω*_j_*. The emitters’ operators will be denoted by $\sigma_{\nu \nu^{\prime }}^{j}=\nu_{j}\nu_{j}^{\prime }$ (ν, ν′ = *g*, *e*), where *e_j_* and *g_j_* stand for the excited and ground state of the *j*-th emitter, respectively. Then, provided that the emitters are locally coupled to specific sites of the photonic bath **r***_j_*, the Hamiltonian of the full system reads as (see Fig. 1A)

$H=H_{B}+\sum_{j=1}^{N}(\omega_{j}\sigma_{ee}^{j}+\sum_{r}g_{{rr}_{j}}\;a_{r}^{†}\sigma_{ge}^{j}+H.c.)$ where *N* is the total number of emitters and *g*_**rr***_j_*_ = *g δ*_**rr***_j_*_, with *g* representing the light-matter coupling strength. This type of light-matter coupling Hamiltonians can describe both the situation where natural or artificial atoms couple to real photonic crystal environments (*10*) and others where superconducting qubits couple to microwave resonator arrays (*22*, *41*–*43*). Besides, it is noteworthy that similar light-matter interaction Hamiltonians can be emulated with purely atomic setups by replacing the role of photons by matter waves (*44*, *45*). The latter is a particularly promising platform to test our predictions given that both the first implementation of such simulated light-matter interfaces (*46*) and Weyl points (*47*) have been recently achieved.

#### Tailoring the Weyl environment

The discrete lattice model that embodies the photonic Weyl environment is given, for definiteness, by a generalization of the proposal described in (*48*), which we design to break inversion and time-reversal symmetries. However, we expect that the conclusions that we derive from this model can be extended to any tight-binding scheme featuring similar dispersive properties, particularly to those hosting a prototypical type I semimetallic phase (*49*–*51*). Furthermore, even if some tilting of the Weyl cones is assumed, the local character of the light-matter coupling might preclude the photonic excitation from leaking into the bulk modes, thereby enabling the generalization of the obtained results to this class of systems too.

A real space representation of the considered model is outlined in Fig. 1B. It is useful to present it as a set of bidimensional layers, consisting of square lattices, stacked along the *z* axis. The sites within each layer are connected through nearest- and next-nearest-neighbor interactions (with amplitude *J* and *J*′, respectively), whereas interlayer couplings occur solely between vertically aligned sites (with amplitude *J_z_* = *J*). To obtain the Weyl phase, we impose a nontrivial phase pattern that involves the definition of a two-site unit cell. The latter introduces a sublattice degree of freedom that acts as a “pseudospin.” In Fig. 1 (B and C), the sites of what we define as the *A* and *B* sublattices are represented by black and white shallow cylinders, respectively. Last, we include a staggered mass term *m*, which creates an onsite energy offset between the modes belonging to the two different sublattices. Assuming periodic boundary conditions, the Hamiltonian matrix can be written in reciprocal space as follows (see details in the Supplementary Materials)

${\bar{H}}_{B}(k)=\omega_{W}1+d(k)\text{σ}$ where **σ** = (σ*_x_*, σ*_y_*, σ*_z_*), with σ_*x*,*y*,*z*_ representing the Pauli matrices, and **d**(**k**) is a **k**-dependent vector whose components are given by

$$\left\{ \begin{array}{c} d_{x}(k)=-J[\cos(k_{x}a+\varphi )+\sin(k_{x}a)+2\cos(k_{y}a)],\\ \;d_{y}(k)=+J[\sin(k_{x}a+\varphi )+\cos(k_{x}a)],\\ d_{z}(k)=-m-2J\cos(k_{z}a)+4J^{\prime }\sin(k_{x}a)\sin(k_{y}a). \end{array}\right.$$

Here, *a* denotes the distance between nearest neighbors, and we define φ as the complex phase picked up by the excitation when it jumps from a *B* site to an *A* site in the negative *x* direction. In the following, to center the discussion, we fix φ = 0 unless explicitly specified.

##### Phase diagram

The positions of the Weyl points in the Brillouin zone are obtained by solving ∣**d**(**k**)∣ = 0. To delineate the different regions that comprehend the phase diagram shown in Fig. 1D, we study the moving and merging of these Weyl nodes as we sweep the parameter’s space spanned by *m* and *J*′. The green shadowed area occupying the lower left corner of the phase diagram corresponds to a Weyl semimetallic phase featuring two pairs of Weyl points (WSM_2_). This phase can evolve into a different type of Weyl semimetal, which is characterized by the presence of a single pair of band touching points (WSM_1_). The WSM_1_ phase emerges in between a normal insulator (BI) and a quantum (anomalous) Hall insulator (QHI), as expected (*52*). The topological characterization of these gapped phases can be done using a dimensional reduction strategy (*49*, *53*). The latter consists in calculating the Chern number of an effective 2D model stemming from treating *k_z_* as a free parameter in the matrix Hamiltonian defined by Eq. 4. We have found that the topological invariant of the QHI phase is *C* = ± 1 for all values of *k_z_*, whereas the Chern number in the BI phase is always zero (see details in the Supplementary Materials).

##### Emergence of the Fermi arcs

As a natural consequence of the bulk-edge correspondence, when the photonic environment is prepared in the Weyl semimetallic phase, we expect topologically protected edge states to show up. To characterize the surface modes of our model, a slab-like geometry is investigated. To implement that, we impose open boundary conditions along some specific spatial direction while preserving the discrete translation symmetry within the remaining ones. For the sake of conciseness, we consider a slab featuring an infinite size along the *z* and *x* + *y* directions but a finite width along the *x-y* direction, as shown in Fig. 1C. The boundaries of such slab correspond to two planes, which we choose to be composed by sites belonging to the *A* sublattice and we refer to as the (010) and the $\left(0\bar{1}0\right)$ facets. Note that, for convenience, we have introduced a coordinate system rotated π/4 around the *z* axis. In this transformed basis, the lattice vectors are $a_{1}=\sqrt{2}a\;{\hat{e}}_{∥}$, $a_{2}=\sqrt{2}a\;{\hat{e}}_{⊥}$, and $a_{3}=a\;{\hat{e}}_{z}$, where ${\hat{e}}_{∥/⊥}=\frac{1}{\sqrt{2}}({\hat{e}}_{x}\pm {\hat{e}}_{y})$ and ${\hat{e}}_{\alpha }$ denotes a unit vector in the α direction.

The periodicity of the lattice along the *z* and ∥ directions can be exploited to exactly diagonalize the bath Hamiltonian with the selected boundary conditions. To do so, we consider an extended unit cell [see the gray shadowed area in Fig. 1C (left)] and define the surface lattice vectors as $a_{1}^{s}\equiv a_{1}$ and $a_{2}^{s}\equiv a_{3}$. Equipped with these two elements, one can invoke the Bloch theorem to bring *H*_B_ to its diagonal form. The **k**-space’s primitive cell of this quasi-2D model is precisely the surface Brillouin zone. There, the Fermi arcs appear as the *E_n_*(**k**) = ω*_W_* equifrequency contours of the calculated band structure [see inset of Fig. 1D], where *E_n_*(**k**) denotes the *n*-th band.

The Fermi arcs associated with the $\left(0\bar{1}0\right)$ and (010) facets are shown in Fig. 1 (E to G) for three different points of the phase diagram (gray solid and dotted lines, respectively). As seen, they stand as open curves that connect the projections over the surface Brillouin zone of Weyl points with different chirality. The displayed color maps depict the surface Berry curvature associated with the edge band Ω_eb_(**k**), which corresponds to the red colored band in the inset of Fig. 1D. These maps show that, for the (II) and (III) configurations, there are some regions along which the surface Berry curvature exhibits a divergent behavior (*54*). These “hot lines” are associated to areas in reciprocal space where the localization of the wave function changes drastically (see details in the Supplementary Materials).

### Probing Fermi arc surface states via spontaneous emission

As mentioned in the Introduction, the absence of a Fermi energy in the photonic setting makes the detection of the Fermi arcs impossible to be directly extrapolated from the electronic context. The first measurements used angle-resolved transmission to detect the Fermi arcs in photonic systems (*10*, *55*), whereas more refined experiments using classical local probes and near-field scanning measurements (*56*–*59*) have been able to provide better visualization of these modes. In this section, we show that Fermi arc light-matter interfaces represent an outstanding alternative way of probing and imaging the Fermi arcs by monitoring the emitters’ spontaneous emission. For that, we first show that a single emitter locally coupled to the edge of a Weyl system naturally excites surface modes into the bath. Then, we show that if one couples, not to one but to many emitters, and monitor their spontaneous emission far from the surface, one can have a visualization of the Fermi arcs of the bath they are coupled to.

#### Launching Fermi arc surface modes with a single emitter

**Fig. 2.**
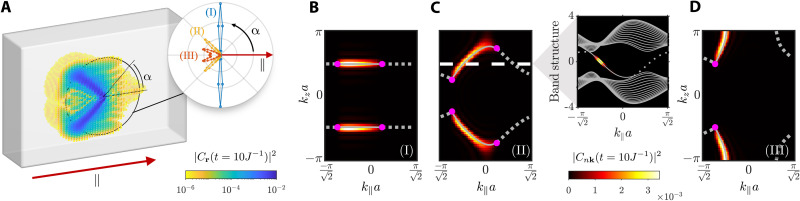
Probing the surface modes of the Weyl system via local light-matter coupling with a single emitter. (**A**) Photonic component of the overall-system wave function at *tJ* = 10 for the configuration marked by (II) in the phase diagram of Fig. 1D. The system is prepared with the emitter in its excited state and coupled to the central site of the $\left(0\bar{1}0\right)$ surface. The inset shows the probability of finding the photonic excitation at **r** = R( cos α, sin α), with R/*a* ≈ 20, after a measuring time of *tJ* = 60, for each of the three cases considered in the phase diagram of Fig. 1D. (**B** to **D**) Distribution of the Bloch modes’ population at *tJ* = 10 for the three considered cases. In all the calculations, the light-matter coupling strength is *g*/*J* = 0.5. A finite slab of width $w/a=16\sqrt{2}$ is used. The dimensions of the $\left(0\bar{1}0\right)$ facet in the ∥ and *z* directions are $L_{∥}/a=63\sqrt{2}$ and *L_z_*/*a* = 63, respectively. To compute the inset of (A), the slab is enlarged in the vertical direction (*L_z_*/*a* = 255) to avoid reflection effects with the top and bottom edges of the facet.

Let us start by considering a single emitter, prepared in its excited state and locally coupled to one of the sites in the lattice’s boundary. The photonic excitation is injected in the system via spontaneous emission, and its propagation through the reservoir can be studied using an exact treatment. The latter implies solving the Schrödinger equation for large finite baths (*30*). For that, we introduce an overall–wave function ansatz of the form (setting the number of emitters *N* = 1)

$$|\text{Ψ}(t)\rangle =\left[\sum_{j=1}^{N}C_{j}(t)\sigma_{eg}^{j}+\sum_{r}C_{r}(t)a_{r}^{†}\right]|{\text{Ψ}}_{\mathrm{vac}}\rangle$$ where ∣*C_j_*(*t*)∣^2^ and ∣*C*_**r**_(*t*)∣^2^ yield the population of the *j*-th emitter and of the bosonic mode localized at position **r**, respectively, and ∣Ψ_vac_⟩ ≡ ∣*g*_1_⋯*g_N_*; vac⟩, with ∣vac⟩ the electromagnetic vacuum. Since our calculations are performed in a finite system, a specification of the photonic lattice’s shape is needed. We consider the one shown in Fig. 2A, which resembles (modulo its finite size) the slab described in the previous section. Then, the emitter, which we locate in the center of the $\left(0\bar{1}0\right)$ surface, is tuned to the Weyl frequency.

Under free evolution, the emitter relaxes to its ground state and the photonic excitation is transferred to the Weyl environment. The latter spreads out, mostly, among the sites comprising the boundary wherein the emitter is placed. This demonstrates a preferential coupling to the surface modes of the bath. Despite being extended over the 2D facet of the system, the photonic excitation propagates mostly in the forward directions, ultimately related to the fact that the considered lattice breaks time-reversal symmetry. Since this resembles what happens in 1D chiral quantum optical settings (*34*), we will refer to these excitations as chiral surface modes. In Fig. 2A, we show the photonic population at each lattice site for the temporal frame *tJ* = 10, with *H*_B_ prepared in the configuration marked by (II) on the phase diagram of Fig. 1C and assuming that *g*/*J* = 0.5. There, the presence of two channels of highly collimated emission oriented in the upward and downward directions reflects the mirror symmetry displayed by the Weyl bath along the *z* = 0 plane. This condition, together with the chiral behavior experienced by the photonic excitation, yield a V-shaped emission profile with no analog in locally coupled light-matter interfaces (*60*), nor in 2D photonic crystals exhibiting supercollimation (*61*), because, there, the chiral nature of the photonic environment is absent. The yellow line in the inset of Fig. 2A shows the probability of finding the excitation at a given angle α within a circle of radius R/*a* ≈ 20, centered at the emitter’s position, after a measuring time of *tJ* = 60. This calculation is repeated for configurations (I) and (III), yielding the blue and orange plots, respectively, showing how one can control the emission patterns through the bath parameters.

If we restrict the study of the system’s dynamics to time values such that *tv* < *L*, where *v* ∼ *Ja* is the average velocity at which the excitation propagates through the bath and *L* accounts for the linear extent of the surface wherein the emitter is placed, reflection effects at the facet’s borders can be neglected. In that case, the results stemming from solving the Schrödinger equation in the finite-sized lattice do not differ from those that one would have obtained if an infinite slab had been considered. Thus, it is legitimate to introduce an alternative representation in which the evolution of the photonic component is described by the time-evolving population of the ensemble of Bloch modes that diagonalize *H*_B_ when the slab-like boundary conditions are imposed (see the Supplementary Materials). The time-dependent population of each Bloch mode can be calculated as follows

${|C_{nk}(t)|}^{2}={|\langle \psi_{nk}|\text{Ψ}(t)\rangle |}^{2}$ where ∣ψ_*n***k**_⟩ is the Bloch state associated to the *n*-th band and the quasi-momentum **k** = (*k*_∥_, *k_z_*). Pretty much in the same way as in the electronic context, one can build some intuition upon the excitation’s dynamics by adopting a semiclassical description (*62*). Within this picture, one can relate the speed and direction of the propagating excitation to the group velocities of the Bloch modes that couple to the emitter.

The population of Bloch modes arising from the photonic profile presented in Fig. 2A is shown in Fig. 2C. Together with that, we plot the projections of the Weyl points over the surface Brillouin zone (magenta dots). We observe that the excited modes outline the shape of the Fermi arcs corresponding to the surface in which the emitter is placed (see the straight gray lines highlighted in the main panel). The inset displays a cut of the band structure for *k_z_a* = π/2, where the colored points reveal the energy distribution of the excited modes. Similar treatment is performed for the configurations (I) and (III), obtaining equivalent mappings as demonstrated in Fig. 2 (B and D, respectively). To understand why this imaging occurs, we first note that, as long as we remain in the regime where *g*/*J* < 1, the modes playing the most relevant role in the dynamical process are those in resonance with the emitter’s frequency. In our case, as we are considering a type I Weyl semimetal and tuning the emitter to the Weyl frequency, we can ensure that the dynamics will be dominated by the Bloch states comprising the Fermi arcs, namely, because they are the ones whose associated energy coincides with ω*_W_*. Besides, the fact that the emitter acts as a local probe in real space translates into a strong nonlocal character of the coupling in **k**-space, which means that all the modes comprising the Fermi arcs are, in principle, homogeneously excited. Nonetheless, we must take into account that, because leakage of the photonic population to the bulk is strongly suppressed whenever the emitter is in resonance with the Weyl frequency, only the Fermi arcs corresponding to the specific boundary where the emitter is embedded can be actually probed.

##### Transforming from real to reciprocal space representation using time-of-flight pictures

**Fig. 3.**
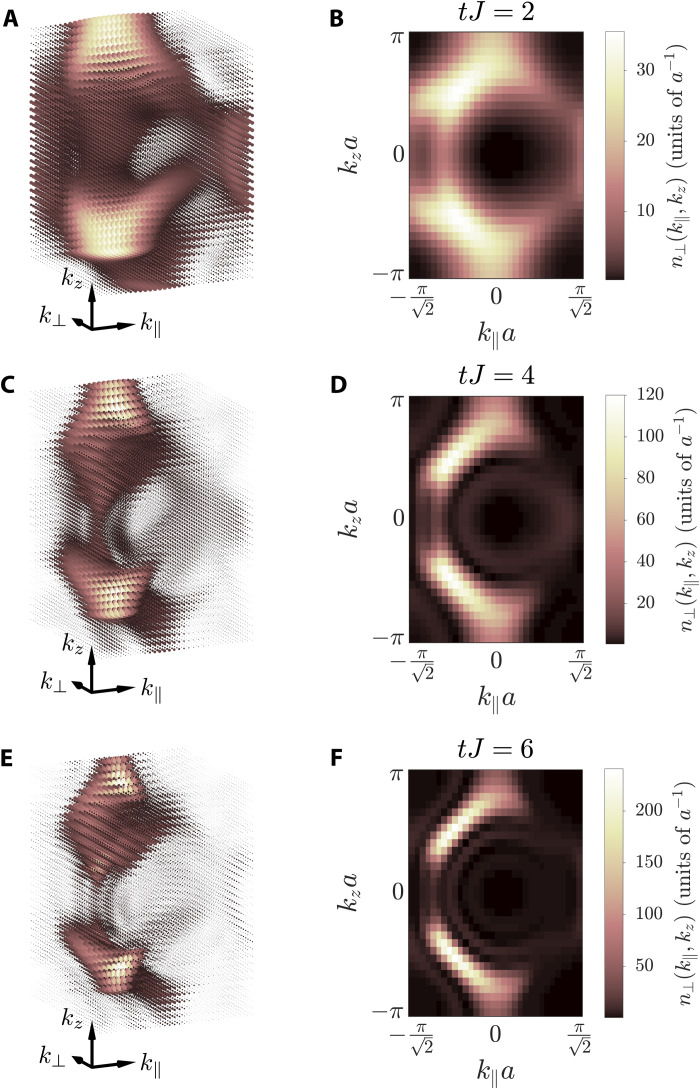
Fermi arcs visualization via time-of-flight imaging of the Weyl photonic environment coupled to an initially excited emitter. We consider a finite lattice constituted by 31 sites in the ∥ and ⊥ directions and 61 sites in the *z* direction (see Fig. 1C), prepared in the configuration marked by (II) in the phase diagram of Fig. 1D, and harboring an initially excited emitter coupled to the center of the $\left(0\bar{1}0\right)$ facet, with *g*/*J* = 0.5. (**A**, **C**, and **E**) 3D momentum distribution *n*(**k**) of the photonic modes excited by the emitter for released times of *tJ* = 2, 4, and 6, respectively. (**B**, **D**, and **F**) Projection of the momentum distribution obtained in (A, C, and E) along the *k*_⊥_ direction.

The dynamical behavior illustrated in Fig. 2A shows that the emitter is acting as a local probe launching surface modes over the photonic structure (*15*, *55*, *56*, *59*). A natural question to ask is whether there is a way of recovering the “Fermi arc picture” once the excitations are transferred into the bath degrees of freedom. Here, we want to illustrate that, for the case of the emulated light-matter interfaces using ultracold atoms (*44*–*46*, *63*), in which the photons are nothing more than matter waves propagating through an optical lattice, there is a straightforward way of recovering them. The idea consists in, once the excitation has been launched, removing the optical traps so that the matter waves are released. This is the method known as time-of-flight imaging (*64*), and it is used routinely in cold atom experiments (*65*). After switching off the trap, the density distribution of the propagating atomic cloud can be shown to be related to the following momentum distribution (see the Supplementary Materials):

$n(k)=\sum_{j,j^{\prime }}e^{ik(r_{j}-r_{j^{\prime }})}\langle \text{Ψ}(t)|a_{r_{j}}^{†}a_{r_{j^{\prime }}}|\text{Ψ}(t)\rangle$ which can be measured through resonant absorption imaging after releasing the matter waves. In Fig. 3 (left), we plot the 3D momentum distribution *n*(**k**) associated with the photonic population featured by the Weyl environment, prepared in the configuration marked by (II) in Fig. 1C, for several de-excitation times in the different rows, whereas in the right column, we plot the corresponding column-integrated momentum distribution along the ⊥ direction: *n*_⊥_(*k*_∥_, *k_z_*) = ∫ ‍ *dk*_⊥_ *n*(**k**). In these panels, we observe that, as the emitter excitation decays completely into the environment, the Fermi arc shape found using the formal mapping defined in the previous section emerges [compare Fig. 3F with Fig. 2C].

#### Fermi arc imaging through many emitters’ spontaneous emission

**Fig. 4.**
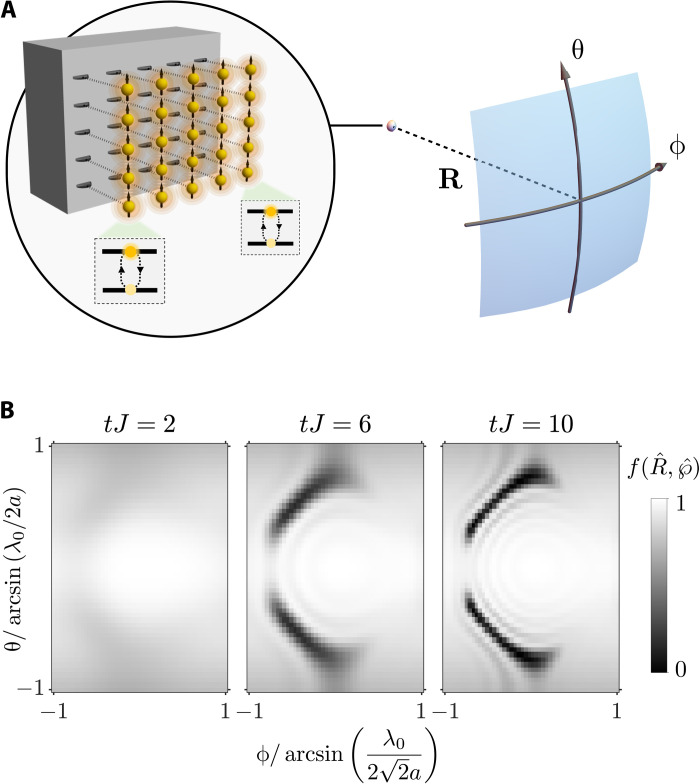
Radiation emanating from an emitter array coupled to the boundary of the Weyl environment. (**A**) Sketch of the considered situation: The radiation stemming from an array of emitters, locally coupled to the sites forming the $\left(0\bar{1}0\right)$ facet of the Weyl lattice, is collected in the far-field as a function of the angular variables θ and ϕ. The light-matter coupling strength *g*, polarization direction $\hat{℘}$, and transition’s wavelength λ_0_ are assumed to be the same for all the considered emitters. At the initial time, only the emitter placed in the center of the face is in its excited state, with no photonic excitations in the bath. (**B**) Angular dependence of the radiation pattern obtained for three different time frames provided that the Weyl bath is prepared in the configuration marked by (II) in the phase diagram of Fig. 1D and that *g*/*J* = 0.2, $\hat{℘}=\hat{z}$, and λ_0_/*a* = 1.

A final question regarding the imaging of Fermi arcs is whether there is a method that does not rely on the matter wave nature of the photonic excitation. In this section, we provide a positive answer by showing that, by monitoring the free-space spontaneous emission of a set of emitters attached to the border of the Weyl environment, one can image the Fermi arcs in reciprocal space. To illustrate that, we consider an array of emitters coupled to the $\left(0\bar{1}0\right)$ surface (one emitter per lattice site in the facet), as shown in Fig. 4A. Then, we assume that the central emitter is excited, while the rest are in their ground state. One can show that as the central emitter decays into the bath as surface modes, it will also excite the rest of the atoms, which will eventually decay into the bath as well. If one considers that the emitters not only decay into the bath but also radiate into free-space modes, then one can monitor the formation of the Fermi arcs in real space. In particular, if we assume that the emitters radiate as electric dipoles, the intensity of the light being emitted at position $R=|R|\hat{R}$ is given by ⟨Ψ(*t*)∣**E**^−^(**R**)**E**^+^(**R**)∣Ψ(*t*)⟩. Here, **E**^+^(**R**) stands for the positive frequency component of the electric field that, in the Markovian approximation, reads (*66*)

$$E^{+}(R)=\mu_{0}\omega_{0}^{2}\sum_{j=1}^{N}{G^{\;↔}}_{0}(R,r_{j},\omega_{0})℘\sigma_{ge}^{j}$$ where we assume that all emitter dipoles are equally oriented, with dipole moment $℘=|℘|\hat{℘}$ and resonant frequency ω_0_. Furthermore, provided that the Green’s tensor is solely given by the far-field contribution of the free-space one

${G^{\;↔}}_{0}(R,r_{j},\omega_{0})=\frac{e^{ik_{0}(|R|-\hat{R}r_{j})}}{4\pi |R|}\left[I^{\;↔}-\frac{R⊗R}{{|R|}^{2}}\right]$ where *k*_0_ = 2π/λ_0_, with λ_0_ being the wavelength of the emitters’ transition, it can be shown that

$$\left\langle \text{Ψ}(t)|E^{-}(R)E^{+}(R)|\text{Ψ}(t)\rangle ={\left(\frac{\mu_{0}\omega_{0}^{2}|℘|}{4\pi |R|}\right)}^{2}f(\hat{R},\hat{℘}\right)$$

Here, we have used the overall–wave function ansatz given by Eq. 6. The temporal dependence is hidden in the form factor $f(\hat{R},\hat{℘})$ through the emitter populations *C_j_*(*t*)

$$f(\hat{R},\hat{℘})={\left[(\hat{R}\times \hat{℘})\times \hat{R}\right]}^{2}\sum_{j,j^{\prime }=1}^{N}C_{j}^{*}(t)C_{j^{\prime }}(t)\;e^{ik_{0}\hat{R}\;r_{jj^{\prime }}}$$ with $\hat{R}=\cos\theta \cos\phi \;{\hat{e}}_{⊥}+\cos\theta \sin\phi \;{\hat{e}}_{∥}+\sin\theta \;{\hat{e}}_{z}$ and **r**_*jj*′_ = **r***_j_* − **r**_*j*′_. In Fig. 4B, we plot precisely this form factor for different times provided that the photonic Weyl environment is prepared in the same phase as in Figs. 3 and 2B, showing again the emergence of the Fermi arc signatures in reciprocal space.

To conclude, let us emphasize that the presented approaches provide a suitable theoretical framework to develop protocols in which the nonlinear behavior of the emitters can play a more fundamental role, e.g., emitting correlated light with which one can perform spectroscopy and measure properties inaccessible by classical probes with the same intensities (*67*).

### Photonic Fermi arc surface states as robust quantum links

After having shown in the previous section that emitters at the edges couple efficiently to the topological surface modes associated with the Fermi arcs, here, we illustrate how to harness them to induce robust quantum links between the emitters. For that, we exploit one of the most notable features of such surface modes, that is, that they can lead to NR at the hinge that separates two different facets of the Weyl system (*15*). Such effect, predicted originally by Veselago (*68*) between materials with different “rightnesses,” is triggering a lot of theoretical and experimental activity (*15*, *69*–*78*) because of its potential uses, for example, to obtain perfect lensing (*79*). However, most of the applications so far have focused on the (semi)-classical regime. Here, we show how to exploit such phenomena to obtain a robust quantum link between emitters that can be harnessed for both perfect quantum state transfer (*36*) and to induce maximal entanglement between the emitters in several ways. To show that, we divide this section into three parts:

1) We determine the system’s parameters to enable NR in the Weyl system and optimize the bath configuration to maximize the coupling between emitters coupled to its edges.

2) We consider the collective dynamics of two emitters placed at consecutive facets of a large-enough system so that revival effects in the initially excited emitter do not occur. In that case, we demonstrate that, under certain conditions, the chiral propagation of the surface modes together with its NR at the hinge make the photonic excitations behave as a perfect 1D, chiral channel (*34*).

3) We consider the opposite limit where multiple refractions occur at the system’s hinges. There, we show how, engineering the system properly, the light emitted from an emitter can arrive at the same point, forming effective 1D cavity modes. We demonstrate that such effective cavity modes induce perfect coherent exchanges between the emitters that can maximally entangle them.

#### Optimizing the Weyl system for NR

**Fig. 5.**
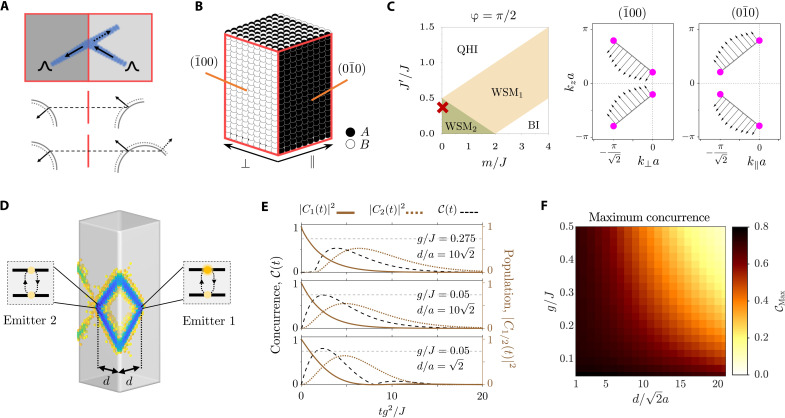
Utilization of the surface Weyl states as an ideal quantum link by exploiting the NR of the photonic excitation at the system’s hinges. (**A**) Single mode picture of the NR process occurring on the surface of a Weyl system. Middle and bottom: The equifrequency contours associated with a system without and with back-propagation, respectively. (**B**) Boundary conditions required to produce NR in the hinge formed between the $\left(0\bar{1}0\right)$ and the $\left(\bar{1}00\right)$ facets. (**C**) Phase diagram of the lattice model for ϕ = π/2 (see definition in Eq. 5). The inset shows the Fermi arcs corresponding to the $\left(\bar{1}00\right)$ and $\left(0\bar{1}0\right)$ faces provided that the bath is prepared in the configuration marked in the phase diagram with a red cross. Black arrows represent the group velocities associated with a set of selected **k**-points belonging to the Fermi arcs in arbitrary units. (**D**) Investigated scenario in which two quantum emitters coupled to adjacent facets of the Weyl bath and separated some distance *d* from the corner formed between the two considered facets. (**E**) Population dynamics and concurrence for different values of the light-matter coupling strength and distance between the emitters. (**F**) Maximum concurrence achieved as a function of the distance between emitters and the light-matter coupling strength.

In the discussion accompanying Fig. 2, we already report on how an emitter tuned to the Weyl frequency preferentially excites the surface modes associated with the Fermi arc dispersions. Within a single facet, because of the mirror symmetry of the bath, an emitter always launches its excitations on two channels with opposite vertical component of the group velocity (see Fig. 2A). This chiral, multichannel emission could be used, e.g., for multiplexing quantum information in different directions (*17*, *18*), thereby enabling the design of quantum link architectures that cannot be obtained with pure 1D setups. In this section, however, we are interested in the possibility of refocusing these channels onto a second emitter at the contiguous edge (see Fig. 5D). For that, we exploit the NR occurring at the system’s hinges (*15*, *69*–*73*).

A schematic view of the refraction process in the interface formed between two adjacent facets of the Weyl bath is depicted in Fig. 5A, where we represent an incoming Bloch state approaching from the right to one of the lattice’s hinges. The group velocity of the targeted mode is given by a black arrow. At the interface, frequency and momentum parallel to the intersection are conserved (*15*), which implies that NR occurs if the tangential component of the group velocity changes its sign. Within this framework, we can also identify the conditions to inhibit reflection, which entail the absence of resonant back-propagating modes in the first surface (compare the middle and bottom panels in Fig. 5A). A preliminary examination of the direction of growth of equifrequency contours associated with the $\left(0\bar{1}0\right)$ and the $\left(\bar{1}00\right)$ surfaces unveils that NR can be achieved between these two facets provided that the first one is composed by sites belonging to the *A* sublattice, whereas the second one is composed of sites belonging to the *B* sublattice, as shown in Fig. 5B.

On top of that, to maximize the focusing onto a single spot, one must account for the fact that the group velocities characterizing the Bloch states of a given Fermi arc do generally feature slightly different propagation directions. This causes each mode in the Fermi arc to undergo a distinct refraction angle, hindering the focalization of the photonic component on the second surface. To circumvent this problem, we explore the configurations’ space of *H*_B_, examining different values of the parameter φ defined in Eq. 5. We find that the optimal situation is obtained by choosing φ = π/2, instead of the φ = 0 value used for Figs. 1 to 3. The phase diagram as a function of *m* and *J*′ for this value of φ = π/2 is displayed in Fig. 5C. The Fermi arcs of the $\left(0\bar{1}0\right)$ and the $\left(\bar{1}00\right)$ facets for the case *m*/*J* = 0 and *J*′/*J* = 0.4 are plotted in the inset. The calculation of the group velocities associated with the **k**-points that span these curves evidences a homogeneous distribution of the direction of propagation of the corresponding Bloch modes. To illustrate that, we plot the group velocities of some selected points in the Fermi arcs using black arrows. In what follows, we fixed this parameters’ configuration and describe the emergence of the two working regimes explained in the introduction of this section. It must be noticed however that, owing to the all-angles NR supported by the Fermi arc light-matter interface (*74*), even in the nonideal cases, the coupling between emitters connected through the surface modes of the Weyl system is notable (see the Supplementary Materials).

To investigate the aforementioned regimes, we will use the setup depicted in Fig. 5D, that is, considering two emitters separated at a distance *d* from the intersection formed between the two studied facets. Besides, we will assume that the emitter at the $\left(0\bar{1}0\right)$ surface is excited, while the other one, in the $\left(\bar{1}00\right)$ surface, is initially in its ground state. After that, we let the system evolve freely and track the populations of both the initially excited and the initially de-excited emitters, which are given by ∣*C*_1_(*t*)∣^2^ and ∣*C*_2_(*t*)∣^2^, respectively. We also study the concurrence C(*t*) as a measure of the two-qubit entanglement (*80*), which for these initial conditions can be shown to be given by (*35*, *81*)

$$C(t)=2|C_{1}(t)C_{2}^{*}(t)|$$

There will be two relevant magnitudes that will distinguish the dynamical regimes that we will discuss in the next two sections. One is the expected decay time of the emitters that, within a Markovian regime, will be of the order τ ∼ *O*(*J*/*g*^2^). The other one is the time that an excitation will take to make a round trip within the system, which will be of the order $T_{R}∼\frac{\ell }{Ja}$, with ℓ being the path length followed by the excitations, which will increase linearly with system size, and *Ja* being the typical group velocity that can be obtained in this system. As we will show below, the behavior will be very different when τ ≫ (≪)*T*_R_.

#### Fermi arc surface states as perfect 1D, chiral channels

Let us first consider the limit of very large systems, that is, τ ≪ *T_R_*. This means that the photons will decay from the emitters completely before any re-excitation can occur. This leads to an effective nonunitary dynamics in the emitters because all the population is eventually lost into the bath. We simulate that regime numerically by including local losses as imaginary energies in the (010) and (100) facets, which attenuates any photonic excitation that arrives to them.

Despite the nonunitary nature of the dynamics, the refocusing of the two channels due to the NR at the hinge (see Fig. 5D) ensures that the excitations leaked by the first emitter can be absorbed by the second. This is clearer in Fig. 5E where we plot the population dynamics of the first (second) emitter in solid (dotted) brown lines, together with the associated concurrence C(*t*) in dashed black, for several parameters. There, we observe how the refocusing of the surface modes can induce a transient entangled state between the emitters. This is the effect known as spontaneous generation of entanglement, which has been recently studied in different contexts (*82*–*87*), including 1D chiral waveguides (*35*). In the latter work, it was shown that, for a perfect Markovian chiral quantum optical channel, the maximum transient entanglement that can be achieved is 2/*e* ≈ 0.736. This is the value that we mark with the horizontal, dotted, gray line in Fig. 5E showing how we can reach the maximum in the situations where the emission is chiral and quasi-1D. For the smallest distance shown (see the bottom panel), the concurrence can go even beyond the ideal limit. This is attributed to the fact that, in that case, the emission does not have space to feature a quasi-1D nature, yielding values slightly above it. Last, to complete the characterization, in Fig. 5F, we make a contour plot of the maximum transient value of concurrence for different corner-to-emitter distances *d* and *g*/*J* ratios, observing how one can approach the ideal limit for a wide range of configurations.

Beyond the intrinsic interest of such spontaneous generation of entanglement, the most important value of the results illustrated by Fig. 5 (D to F) is that they prove that Fermi arc surface modes can behave as a perfect 1D channel. This opens up the use of all the machinery developed for such systems (*34*). For example, one can increase the value of entanglement by coherently driving the emitters with staggered frequencies (*37*–*39*). In those works, it was shown how the combination of staggered drivings plus chiral quantum optical channels can lead to the formation of many-emitters entangled steady states, where the concurrence can approach its maximum value, i.e., C(*t* → ∞) = 1. Beyond two-level emitters, it is also well known that, by using Λ-transitions controlled by Raman lasers, chiral quantum channels can be used to obtain deterministic quantum state transfer (*36*).

#### Coherent exchanges induced by effective cavity modes

**Fig. 6.**
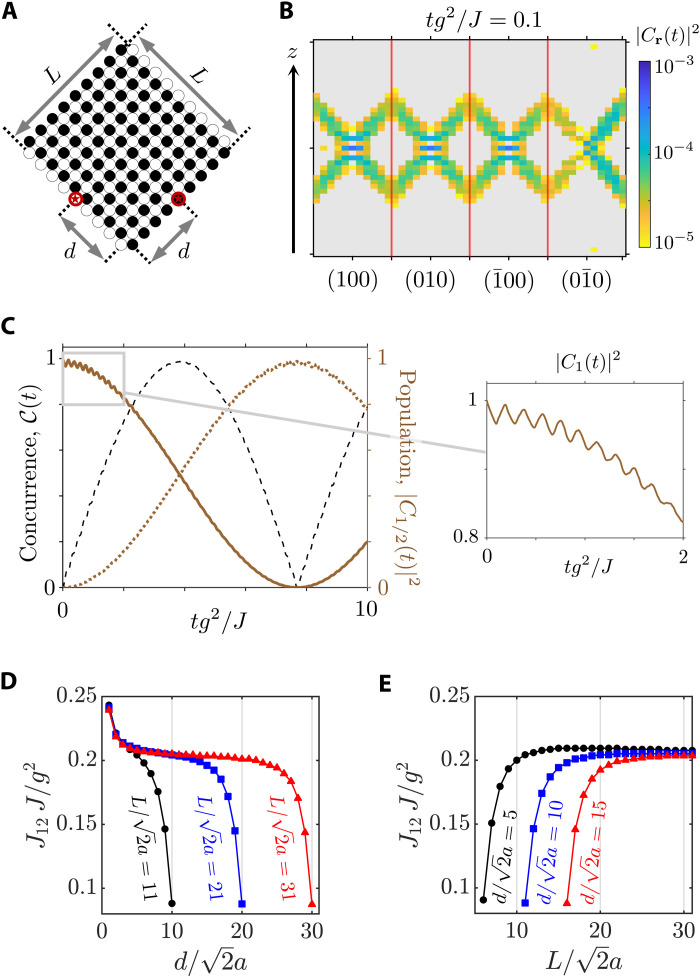
Emergence of an effective off-resonant cavity in the small system’s size limit. (**A**) Top view of the finite lattice model used to generate the effective cavity. Adjacent facets are composed of sites belonging to different sublattices (*A* and *B*). By imposing equally sized faces, we ensure the efficient revival of the considered emitters. (**B**) Photonic population of the bosonic modes belonging to the four different facets in the lattice model for some selected de-excitation time. (**C**) Temporal evolution of the initially excited (solid brown) and initially de-excited (dotted brown) emitters. The dashed black line denotes the calculated concurrence. The inset shows a detailed view of the small oscillations observed at short times, which are related to the time that the excitation spends to perform a complete round trip. (**D**) Frequency of the large oscillations *J*_12_ as a function of the corner-to-emitter distance *d*, for three different system’s sizes $L/\sqrt{2}a=11$, 21, and 31 (black dots, blue squares, and red triangles). (**E**) Oscillation frequency *J*_12_ as a function of the system’s size *L*, for three different corner-to-emitter distances $d/\sqrt{2}a=5$, 10, and 15 (black dots, blue squares, and red triangles).

In the small system’s size limit, that is, when *T_R_* ≪ τ, the occurrence of revivals due to the re-excitation of the emitters cannot be avoided. Rather than being a hindrance, we will show now how NR can turn these revivals into a resource. The key point is that, if one designs the system appropriately, as depicted in Fig. 6A, the photonic excitations circulate around the system arriving, eventually, at the position of the emitter from where they were launched. Again, this is possible because of the NR taking place at the system’s hinges, which guides the propagating ray through a confined, braid-shaped path. An example of that emission pattern is shown in Fig. 6B where we plot a snapshot of the bath population in the facets for a particular time, showing the traces of the focusing and refocusing of light.

This light behavior leads to a radically different dynamics of the emitters. An example of that is shown in Fig. 6C, where we plot the excited state population of the initially excited (de-excited) emitter in solid (dotted) brown. We observe that, differently from the previous situation, the emitters feature perfect coherent exchanges at a distinct frequency that we denote as *J*_12_. We also observe small amplitude oscillations, which are zoomed in the right inset panel, whose frequency can be directly linked to the time it takes for the photonic excitations to perform a complete round trip. The perfect coherent oscillations at frequency *J*_12_ allow the emitter to reach the maximal entanglement of C(*t*) ≈ 1 in the transient regime (see dashed black line), overcoming the limitations of the chiral dissipative channels (*35*).

The intuition on why these coherent oscillations appear is that, thanks to the NR, the photonic excitation undergoes a closed loop, creating an effective 1D cavity that is able to transfer excitations off-resonantly between the emitters (*88*). To confirm this intuition and obtain further insight, we plot, in Fig. 6D, the frequency of the oscillations *J*_12_ as a function of the corner-to-emitter distance for several system sizes. There, we observe that, disregarding the finite size effects that appear when the emitters are located close to the system’s hinges, *J*_12_ tends to be constant with the distance. This is clearer in the plateau obtained for *J*_12_ for the larger system size (in red triangles). Note that this is what is expected for off-resonant cavity couplings because they typically mediate infinite-range interactions (*88*). Apart from that, in Fig. 6E, we plot the opposite situation, that is, we fix several distances between emitters and study the dependence with system size. There, we observe how *J*_12_ also tends to the same constant value for large system sizes. This can also be explained in terms of this effective cavity picture. Typically, off-resonant cavity couplings scale as $J_{12}∼g_{e}^{2}/{\text{Δ}}_{e}$, with *g_e_* being the coupling of the emitter to the effective cavity mode and Δ*_e_* being its detuning. In these setups, the coupling strength always scales with the size of the cavity as $g_{e}\propto 1/\sqrt{\ell_{c}}$. Besides, because the emitter’s energy is fixed, the only dependence with system size in the detuning is that of the energy of the cavity modes, which are spaced by ω*_n_* = *nv_g_*/ℓ_c_ (*88*, *89*). Thus, the dependence with system size in *J*_12_ vanishes, which is why all lines of *J*_12_ tend to a constant value in Fig. 6E.

**Fig. 7.**
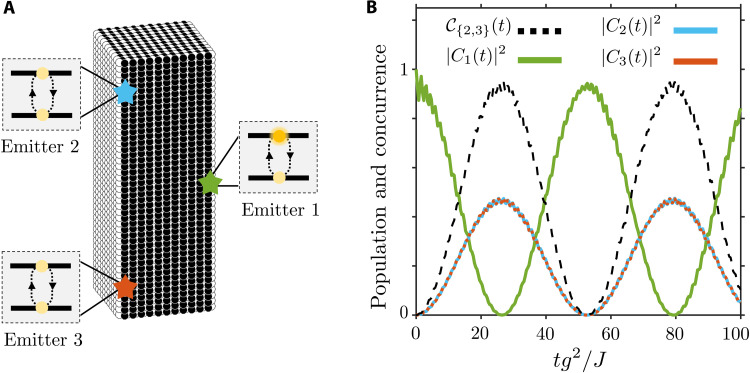
Three-emitter entanglement protocol. (**A**) Real space configuration of the system. (**B**) Population dynamics of the initially excited emitter (solid green line) and of the initially de-excited emitters (dashed blue and orange lines). The concurrence calculated for the two initially de-excited emitters, labeled as the emitters 2 and 3, is plotted using a dashed black line. The light-matter coupling and system’s size are assumed to be *g*/*J* = 0.1 and $L/\sqrt{2}a=21$, respectively.

We have thus shown that the emergence of this effective cavity enables the occurrence of maximum entanglement between distant emitters in the transient regime. Moreover, the versatility offered by the studied system greatly exceeds the one that could be obtained, e.g., by harnessing the 1D chiral channels featured by 2D topological systems. This is because the possibility of obtaining V-shaped emission and quasi-perfect 1D channels within the system’s surface facilitates the coupling between non-collinear emitters, thereby opening exciting avenues toward the design of more complicated quantum emitter architectures. To explicitly show the potential of the Fermi arc light-matter interfaces, we design a three-emitter protocol that cannot be implemented with purely 2D setups. In particular, we show that perfect entanglement between two initially de-excited emitters can be obtained thanks to the V-shaped emission pattern appearing on the surface of the Weyl system. For that, we place the emitters on the system’s boundary, forming a triangular configuration, as displayed in Fig. 7A. The initially excited emitter is located in one of the corners of the lattice and tuned to the Weyl frequency. The photonic excitation is launched following the aforementioned V-shape. Then, we place two additional emitters at the contiguous lattice’s corner. Their positions are chosen so that they couple to different branches of the light beam. The latter corresponds to two perfectly equivalent channels, each of which carries half of the initial excitation. This condition yields an almost perfect state exchange between the initially excited emitter and the initially de-excited ones, which are symmetrically populated as shown in Fig. 7B. The calculation of the concurrence for the initially de-excited emitters confirms the emergence of an almost perfect transient entanglement regime between them. This result demonstrates that the spatial separation of the photonic excitation in the 2D surface of the Weyl system can be harnessed to devise more complicated emitter connectivities while maintaining the desirable properties of simpler systems, e.g., the formation of 1D chiral channels.

## DISCUSSION

Summing up, we have characterized the behavior of Fermi arc light-matter interfaces, discovering several remarkable phenomena. First, we have demonstrated that the studied platforms can be used to image the Fermi arcs in unconventional ways, by monitoring the free-space spontaneous emission of the considered emitters. We have shown how to engineer the coupling to the surface modes so that they behave as a robust quantum channel in both a dissipative and coherent regime. Although we illustrate this behavior by studying the spontaneous formation of two-qubit entanglement between a pair of emitters, our results immediately open up their use for designing quantum state transfer protocols (*36*) and for obtaining nontrivial entangled steady states (*37*–*39*), among other applications. The latter evidence the great potential of the Fermi arc surface states to be harnessed for quantum technological applications. Furthermore, we provide an alternative framework for the exploration of these topological states through their interaction with optically active emitters, uncovering phenomena with no analog in fermionic systems, which can be extended to study other topological surface states (*90*–*92*).

## MATERIALS AND METHODS

All real space simulations follow from the resolution of the Schrödinger equation for the Hamiltonian given in Eq. 3 within the single excitation subspace. For that, we calculate the action of the exponential of the Hamiltonian matrix over the initial state of the system (*30*). The latter is assumed to be the one in which a single emitter is in its excited state and there are no excitations present in the photonic bath.
