# Supplementary material for: Probing and harnessing photonic Fermi arc surface states using light-matter interactions
Source: Sci Adv. 2023 May 31;9(22):eadf8257. doi: 10.1126/sciadv.adf8257 (PMC10413654; doi:10.1126/sciadv.adf8257)
Supplement: Supplementary file 1 — Supplementary Text Figs. S1 to S4 [file sciadv.adf8257_sm.pdf]

Science Advances
AAAS

Supplementary Materials for

Probing and harnessing photonic Fermi arc surface states using light-matter interactions

Iñaki García-Elcano *et al.*

Corresponding author: Iñaki García-Elcano, innaki.garciae@uam.es

Sci. Adv. **9**, eadf8257 (2023)
DOI: 10.1126/sciadv.adf8257

This PDF file includes:

Supplementary Text
Figs. S1 to S4

1 Lattice model

In this section, we provide further details on the lattice model that is used to mimic the photonic Weyl environment.

The different bosonic modes that comprise the reservoir are positioned following a cubic geometry, where the corresponding lattice constant a denotes the minimum separation between sites and is taken as the unit length throughout this section. In order to keep the discussion as general as possible, arbitrary complex hoppings are assumed. This implies that, even in the simplest case in which only nearest-neighbor interactions are implemented, the discrete translation symmetry of the cubic arrangement is broken by the distribution of phases picked up by the excitation when it jumps among the different lattice's sites. Therefore, to take into consideration the magnetic symmetries of the system in all possible situations, a two-sites unit cell must be defined [without loss of generality we chose the unit cell as the gray shadowed area shown in Figs. S1(a-c)]. The lattice vectors are $\mathbf{a}_1 = \hat{\mathbf{e}}_x + \hat{\mathbf{e}}_y$, $\mathbf{a}_2 = \hat{\mathbf{e}}_x - \hat{\mathbf{e}}_y$ and $\mathbf{a}_3 = \hat{\mathbf{e}}_z$, and they are depicted with orange arrows in Fig. S1(a). The position's ensemble spanned by each of the non-equivalent sites in the unit cell composes the A and B sublattices, which are represented in Figs. S1(a-d) by black and white cylinders, respectively. If periodic boundary conditions are imposed in all three spatial directions, the resulting lattice model captures well the dispersive properties of the modes belonging to the bulk of the system. Hereafter, we refer to this particular scheme as the bulk configuration.

Alternatively, one can consider periodicity in only two spatial directions. This choice leads to the formalization of the slab configuration described in the main text, which is used to investigate the edge states of the 3D bath. It is worth noting that, in this case, the direction in which the system is cut and the width of the slab are determined by the specification of both, the surface lattice vectors $\boldsymbol{a}_1^s$ and $\boldsymbol{a}_2^s$, and the unit cell. The latter is, in general, different from

the one defined in the bulk configuration. More precisely, in the slab models, the number of non-equivalent sites belonging to the unit cell, i.e. the number of sublattices N_s, is usually larger than in the bulk configuration.

Irrespective of the selected boundary conditions, the bath Hamiltonian H_{B} can be presented as the sum of four contributions:

$$H_{\mathrm{B}} = H_{\mathrm{os}} + H_{xy} + H_z + H'_{xy}, \tag{S.1}$$

which we express now using a basis of localized bosonic modes. The first term is given by:

$$H_{\mathrm{os}} = \sum_{\mathbf{r}\in A}(\varepsilon - m)a^\dagger_{\mathbf{r}}a_{\mathbf{r}} + \sum_{\mathbf{r}\in B}(\varepsilon + m)a^\dagger_{\mathbf{r}}a_{\mathbf{r}}, \tag{S.2}$$

where ε denotes the bare onsite energy of the bosonic modes and m is a staggered mass term that produces an onsite energy offset between modes belonging to different sublattices. The second and third terms represent the nearest-neighbors intra- and inter-layer interactions, respectively. They read as:

$$\begin{aligned} H_{xy} = -\sum_{\mathbf{r}\in A} & t_1 a^\dagger_{\mathbf{r}} a_{\mathbf{r}+\hat{\mathbf{e}}_x} + t_2 a^\dagger_{\mathbf{r}} a_{\mathbf{r}-\hat{\mathbf{e}}_x} + \cdots \\ & t_3 a^\dagger_{\mathbf{r}} a_{\mathbf{r}+\hat{\mathbf{e}}_y} + t_4 a^\dagger_{\mathbf{r}} a_{\mathbf{r}-\hat{\mathbf{e}}_y} + \text{H.c.}, \end{aligned} \tag{S.3}$$

$$H_z = -\sum_{\mathbf{r}\in A}\left(t_5 a^\dagger_{\mathbf{r}} a_{\mathbf{r}+\hat{\mathbf{e}}_z} + \text{H.c.}\right) - \sum_{\mathbf{r}\in B}\left(t_6 a^\dagger_{\mathbf{r}} a_{\mathbf{r}+\hat{\mathbf{e}}_z} + \text{H.c.}\right), \tag{S.4}$$

where $t_j = |t_j|e^{i\varphi_j}$ is a complex hopping matrix element. The last term accounts for some additional intra-layer next-nearest-neighbours interaction which is included to enrich the phase space of the model. It is described by the following tight-binding Hamiltonian:

$$\begin{aligned} H'_{xy} = &-\sum_{\mathbf{r}\in A}\left(t_7 a^\dagger_{\mathbf{r}} a_{\mathbf{r}+(\hat{\mathbf{e}}_x+\hat{\mathbf{e}}_y)} + t_8 a^\dagger_{\mathbf{r}} a_{\mathbf{r}+(\hat{\mathbf{e}}_x-\hat{\mathbf{e}}_y)} + \text{H.c.}\right)\cdots \\ &-\sum_{\mathbf{r}\in B}\left(t_9 a^\dagger_{\mathbf{r}} a_{\mathbf{r}+(\hat{\mathbf{e}}_x+\hat{\mathbf{e}}_y)} + t_{10} a^\dagger_{\mathbf{r}} a_{\mathbf{r}+(\hat{\mathbf{e}}_x-\hat{\mathbf{e}}_y)} + \text{H.c.}\right). \end{aligned} \tag{S.5}$$

This real space representation of the bath Hamiltonian is especially useful to address finite-sized lattices since it does not relay in any particular boundary condition. Conversely, if discrete

translational invariance is imposed in at least one spatial direction, the problem can be simplified by introducing reciprocal space. To do that, we build a basis of Bloch-like orbitals, defined as follows:

$$|\phi_{\xi_n \mathbf{k}}\rangle = a^{\dagger}_{\xi_n \mathbf{k}} |\mathrm{vac}\rangle, \tag{S.6}$$

where $|\mathrm{vac}\rangle$ denotes the electromagnetic vacuum and $a^{\dagger}_{\xi_n \mathbf{k}}$ is the Fourier transformation of the bosonic operator $a^{\dagger}_{\mathbf{r}}$, which is given by:

$$a^{\dagger}_{\xi_n \mathbf{k}} = \frac{1}{\sqrt{N_{\xi_n}}} \sum_{\mathbf{r} \in \xi_n} e^{i\mathbf{k}\mathbf{r}} a^{\dagger}_{\mathbf{r}}. \tag{S.7}$$

Here, N_{ξ_n} is the total number of sites belonging to the ξ_n-th sublattice and $\mathbf{k}$ is a vector in the first Brillouin zone. Using this relation, H_{B} can be rewritten as a quadratic form:

$$H_{\mathrm{B}} = \sum_{\mathbf{k}} A^{\dagger}_{\mathbf{k}} \, \bar{H}_{\mathrm{B}}(\mathbf{k}) \, A_{\mathbf{k}}, \tag{S.8}$$

where we have defined the row operator $A^{\dagger}_{\mathbf{k}} = (a^{\dagger}_{\xi_1 \mathbf{k}} \cdots a^{\dagger}_{\xi_{N_s} \mathbf{k}})$ and the column operator $A_{\mathbf{k}} = (A^{\dagger}_{\mathbf{k}})^{\dagger}$. Besides, we recognize $\bar{H}_{\mathrm{B}}(\mathbf{k})$ as the matrix of the bath Hamiltonian in the basis of Bloch-like orbitals, whose matrix elements read: $\left[\bar{H}_{\mathrm{B}}(\mathbf{k})\right]_{ij} = \langle \phi_{\xi_i \mathbf{k}} | H_{\mathrm{B}}(\mathbf{k}) | \phi_{\xi_j \mathbf{k}} \rangle$. This is just a $N_s \times N_s$ matrix that can be brought to its diagonal form by identifying the $\mathbf{k}$-dependent unitary transformation $\bar{U}(\mathbf{k})$ that yields:

$$\bar{D}_{\mathrm{B}}(\mathbf{k}) = \bar{U}^{\dagger}(\mathbf{k}) \bar{H}_{\mathrm{B}}(\mathbf{k}) \bar{U}(\mathbf{k}) \tag{S.9}$$

where $\bar{D}_{\mathrm{B}}(\mathbf{k})$ represents a diagonal matrix. In particular, we have that:

$$H_{\mathrm{B}} = \sum_{\mathbf{k}} \tilde{A}^{\dagger}_{\mathbf{k}} \, \bar{D}_{\mathrm{B}}(\mathbf{k}) \, \tilde{A}_{\mathbf{k}}, \tag{S.10}$$

where $\tilde{A}^{\dagger}_{\mathbf{k}} = A^{\dagger}_{\mathbf{k}} \bar{U}(\mathbf{k}) = (\tilde{a}^{\dagger}_{1\mathbf{k}} \cdots \tilde{a}^{\dagger}_{N_s \mathbf{k}})$ and $\tilde{A}_{\mathbf{k}} = \bar{U}^{\dagger}(\mathbf{k}) A_{\mathbf{k}}$. It must be noticed that the non-zero elements of $\bar{D}_{\mathrm{B}}(\mathbf{k})$ account for the bands of the structured bath, i.e., $[\bar{D}_{\mathrm{B}}(\mathbf{k})]_{nn} \equiv E_n(\mathbf{k})$. This is, Eq. (S.10) can be rewritten as:

$$H_{\mathrm{B}} = \sum_{n\mathbf{k}} E_n(\mathbf{k}) \, \tilde{a}^{\dagger}_{n\mathbf{k}} \tilde{a}_{n\mathbf{k}}. \tag{S.11}$$

Moreover, the Bloch state with associated band number n and quasimomentum $\mathbf{k}$ is given by:

$$|\psi_{n\mathbf{k}}\rangle = \tilde{a}^{\dagger}_{n\mathbf{k}}|\text{vac}\rangle, \tag{S.12}$$

or, on the basis of Bloch-like orbitals:

$$|\psi_{n\mathbf{k}}\rangle = \sum_{i=1}^{N_s} u_{in}|\phi_{\xi_n\mathbf{k}}\rangle, \tag{S.13}$$

where $u_{ij} \equiv [\bar{U}(\mathbf{k})]_{ij}$ correspond to the matrix elements of the unitary transformation $\bar{U}(\mathbf{k})$.

Finally, we define the Berry curvature tensor of the n-th band $\Omega^n_{\mu\nu}(\mathbf{k})$, which can be computed by employing the Berry connection (62):

$$\Omega^n_{\mu\nu}(\mathbf{k}) = \frac{\partial \mathcal{A}^n_\nu(\mathbf{k})}{\partial k_\mu} - \frac{\partial \mathcal{A}^n_\mu(\mathbf{k})}{\partial k_\nu}, \tag{S.14}$$

where $\boldsymbol{\mathcal{A}}^n(\mathbf{k}) = i\langle\psi_{n\mathbf{k}}|\frac{\partial}{\partial\mathbf{k}}|\psi_{n\mathbf{k}}\rangle$. Equivalently, using the Bloch-like orbitals basis, $\Omega^n_{\mu\nu}(\mathbf{k})$ can be calculated as follows (omitting the $\mathbf{k}$ dependencies for clarity):

$$\Omega^n_{\mu\nu} = i\sum_{n'\neq n} \frac{(\boldsymbol{u}^*_n \frac{\partial \bar{H}_\mathrm{B}}{\partial k_\mu}\, \boldsymbol{u}_{n'})\,(\boldsymbol{u}^*_{n'} \frac{\partial \bar{H}_\mathrm{B}}{\partial k_\nu}\, \boldsymbol{u}_n)}{(E_n - E_{n'})^2} - (\nu \leftrightarrow \mu), \tag{S.15}$$

where $\boldsymbol{u}_n \equiv (u_{1n}, u_{2n}, \cdots, u_{Nn})^T$ is a $\mathbf{k}$-dependent vector representing the n-th column of the unitary transformation $\bar{U}(\mathbf{k})$ that diagonalizes $\bar{H}_\mathrm{B}(\mathbf{k})$. We remark that the derivation of the band structure and Berry curvature tensor discussed above is valid for both the bulk and slab configurations. In the following, we study each case separately.

1.1 Bulk configuration

When periodic boundary conditions are assumed in all three spatial directions, $\bar{H}_\mathrm{B}(\mathbf{k})$ is given by a $\mathbf{k}$-dependent 2×2 matrix since, in this case, the unit cell contains two non-equivalent sites, i.e., $N_s = 2$. In particular, they correspond to the A and B sublattices. As in the real space representation, we separate $\bar{H}_\mathrm{B}(\mathbf{k})$ into four different contributions:

$$\bar{H}_\mathrm{B}(\mathbf{k}) = \bar{H}_\mathrm{os} + \bar{H}_{xy} + \bar{H}'_{xy} + \bar{H}_z, \tag{S.16}$$

where each contribution reads:

$$\bar{H}_{\text{os}} = \varepsilon \mathbb{1} - m\sigma_z, \tag{S.17}$$

$$\begin{aligned}\bar{H}_{xy} =& - [\ |t_1|\cos(k_x+\varphi_1) + |t_2|\cos(k_x-\varphi_2) + |t_3|\cos(k_y+\varphi_3) + |t_4|\cos(k_y-\varphi_4)\ ]\,\sigma_x \cdots \\ &+ [\ |t_1|\sin(k_x+\varphi_1) - |t_2|\sin(k_x-\varphi_2) + |t_3|\sin(k_y+\varphi_3) - |t_4|\sin(k_y-\varphi_4)\ ]\,\sigma_y,\end{aligned} \tag{S.18}$$

$$\bar{H}_z = - |t_5|\cos(k_z+\varphi_5)\,(\mathbb{1}+\sigma_z) - |t_6|\cos(k_z+\varphi_6)\,(\mathbb{1}-\sigma_z), \tag{S.19}$$

$$\begin{aligned}\bar{H}'_{xy} =& - [\ |t_7|\cos(k_+ +\varphi_7) + |t_8|\cos(k_- +\varphi_8)\ ]\,(\mathbb{1}+\sigma_z)\cdots \\ &- [\ |t_9|\cos(k_+ +\varphi_9) + |t_{10}|\cos(k_- +\varphi_{10})\ ]\,(\mathbb{1}-\sigma_z).\end{aligned} \tag{S.20}$$

Here, we have defined $k_\pm = k_x \pm k_y$ and we have conveniently introduced the identity, $\mathbb{1}$, and the Pauli matrices, $\boldsymbol{\sigma} = (\sigma_x, \sigma_y, \sigma_z)$. It is worth noting that, for this two-band model, $\bar{H}_{\text{B}}(\mathbf{k})$ can be compactly expressed as follows:

$$\bar{H}_{\text{B}}(\mathbf{k}) = d_0(\mathbf{k})\mathbb{1} + \mathbf{d}(\mathbf{k})\boldsymbol{\sigma}, \tag{S.21}$$

with $d_0(\mathbf{k})$ and the different components of $\mathbf{d}(\mathbf{k})$ being continuous functions of $\mathbf{k}$. From Eqs. (S.19) and (S.20), is easy to see that, in order to get a type I semimetal, one must set $|\varphi_5 - \varphi_6| = |\varphi_7 - \varphi_9| = |\varphi_8 - \varphi_{10}| = \pi$ and $|t_5| = |t_6|$, $|t_7| = |t_9|$ and $|t_8| = |t_{10}|$. If those conditions are imposed, one gets $d_0(\mathbf{k}) = \varepsilon$, which we identify with the Weyl frequency, i.e., $\varepsilon \equiv \omega_W$. The band dispersion of the upper and lower bands are given by $E_\pm(\mathbf{k}) = \omega_W \pm |\mathbf{d}(\mathbf{k})|$ which, provided that the system features a semimetallic phase, implies that the position of the Weyl nodes can be obtained by solving $|\mathbf{d}(\mathbf{k})| = 0$. Besides, since the reciprocal space is three-dimensional, the topological properties of the bath are encoded in the Berry curvature vector $\boldsymbol{\Omega}^n(\mathbf{k})$, whose components are related to the Berry tensor by $\Omega^n_{\mu\nu}(\mathbf{k}) = \boldsymbol{\epsilon}_{\mu\nu\xi}[\boldsymbol{\Omega}^n(\mathbf{k})]_\xi$ (62). The latter can be explicitly worked out, yielding:

$$\Omega^\pm_{\mu\nu}(\mathbf{k}) = \mp\frac{\mathbf{d}(\mathbf{k})}{2|\mathbf{d}(\mathbf{k})|^3}\left[\frac{\mathbf{d}(\mathbf{k})}{\partial k_\mu} \times \frac{\mathbf{d}(\mathbf{k})}{\partial k_\nu}\right]. \tag{S.22}$$

Calculating the flux of $\boldsymbol{\Omega}^n(\mathbf{k})$ over a surface enclosing a Weyl point one can determine its corresponding Chern number.

The simplified model employed in the main text can be recovered by particularizing Eq. (S.16) for the set of parameters collected in Table S1. The dispersion relation along the $k_x a = -\pi/4$ plane for the configurations marked by (I), (II) and (III) in the phase diagram of Fig. 1(d) in the main text are represented in the panels (e), (f) and (g) of Fig. S1, respectively. Insets show the Berry curvature (blue arrows) and the Weyl points (magenta dots) for each of the studied configurations.

1.2 Slab configuration

If the discrete translation symmetry is broken along some specific direction while maintained along the remaining ones, the resulting lattice features the form of a slab. A particular example is introduced in the subsection called *Tailoring the Weyl environment* of the main text. In this case, the associated Bloch Hamiltonian is given by a $N_s \times N_s$ matrix featuring the following form:

$$\bar{H}_{\mathrm{B}}(\mathbf{k}) = \left(\begin{array}{|c|c|} \hline \bar{h}_{AA} & \bar{h}_{AB} \\ \hline \bar{h}_{BA} & \bar{h}_{BB} \\ \hline \end{array} \right), \tag{S.23}$$

where the $\bar{h}_{AA(BB)}$ block accounts for the interaction among sites belonging to the $A(B)$ sublattice, whereas the $\bar{h}_{AB}$ and $\bar{h}_{BA} = \bar{h}_{AB}^\dagger$ blocks describe the interactions between sites belonging to different sublattices. Even though the definition of the A and B sublattices is strongly related to the bulk configuration, we maintain such distinction in the construction of the matrix Hamiltonian for the slab configuration. The latter requires to consider the proper ordering of the Fourier-transformed bosonic operators that constitute the row $A_{\mathbf{k}}^\dagger$ and column $A_{\mathbf{k}}$ operators referred to in Eq. (S.8).

The number of non-equivalent sites composing the unit cell N_s determines, in this case, the

width of the slab and, therefore, the dimension of $\bar{H}_{\rm B}(\mathbf{k})$. The matrix elements for each of the considered blocks depend on the direction of the cut. For the example discussed in the main text, each block is given by a tridiagonal matrix, where the non-zero matrix elements read:

$$\left[\bar{h}_{AA(BB)}\right]_{i,i} = -(+)m - [t_{5(6)}\, e^{ik_z} + t_{7(9)}\, e^{i\sqrt{2}k_\parallel} + \text{H.c.}], \tag{S.24}$$

$$\left[\bar{h}_{AA(BB)}\right]_{i,i+1} = -t_{8(10)}, \tag{S.25}$$

$$\left[\bar{h}_{AA(BB)}\right]_{i+1,i} = -t^*_{8(10)}, \tag{S.26}$$

and

$$\left[\bar{h}_{AB}\right]_{i,i} = -t_1\, e^{ik_\parallel/\sqrt{2}} - t_4\, e^{-ik_\parallel/\sqrt{2}}, \tag{S.27}$$

$$\left[\bar{h}_{AB}\right]_{i,i+1} = 0, \tag{S.28}$$

$$\left[\bar{h}_{AB}\right]_{i+1,i} = -t_2\, e^{ik_\parallel/\sqrt{2}} - t_3\, e^{-ik_\parallel/\sqrt{2}}, \tag{S.29}$$

We note that, for the investigated example, an odd number of non-equivalent sites are considered. Also, we chose the $(0\bar{1}0)$ and $(\bar{1}00)$ faces to be composed by sites belonging to the A sublattice. This implies that the dimensions of the $\bar{h}_{AA}$ and the $\bar{h}_{BB}$ blocks are different, namely, we have that the latter are square matrices with dimensions $(N_s+1)/2$ and $(N_s-1)/2$, respectively.

Finally, we point out that the surface Berry curvature associated the slab configuration can be computed using the general expression given by Eq. (S.15). This is what we represent in the color maps of Figs 1(e-g) in the main text. More precisely, we plot the Berry curvature associated with the $n = 17$ band of the considered slab configuration which, provided that we select a unit cell formed by $N_s = 33$ non-equivalent sites, is identified as the edge band, i.e., $\Omega_{\rm eb}(\mathbf{k}) \equiv \Omega^{n=17}_{\parallel z}(\mathbf{k})$ (see details in Section 3 of the Supplementary Materials).

Nearest-neighbours		Next-nearest-neighbours	
Amplitude	Phase	Amplitude	Phase
$\|t_1\| = J$	$\varphi_1 = \varphi$	$\|t_7\| = J'$	$\varphi_7 = 0$
$\|t_2\| = J$	$\varphi_2 = \frac{\pi}{2}$	$\|t_8\| = J'$	$\varphi_8 = \pi$
$\|t_3\| = J$	$\varphi_3 = 0$	$\|t_9\| = J'$	$\varphi_9 = \pi$
$\|t_4\| = J$	$\varphi_4 = 0$	$\|t_{10}\| = J'$	$\varphi_{10} = 0$
$\|t_5\| = J$	$\varphi_5 = 0$		
$\|t_6\| = J$	$\varphi_6 = \pi$		

Table S1: Parameters employed to obtain the lattice model presented in the main text.

2 Topological characterization of the gapped phases.

The topological characterization of the gapped phases found in the phase diagram shown in Fig.1(c) of the main text can be done using a dimensional reduction approach (49,53). The idea is to treat one of the quasi-momentum variables as a free parameter, such that the resulting matrix Hamiltonian corresponds to a 2D system. In that case, the Chern number associated with the upper and lower bands is given by:

$$C_{\pm} = \frac{1}{2\pi} \int_{\mathrm{BZ}} d^2\mathbf{k}\, \Omega^{\pm}(\mathbf{k}). \tag{S.30}$$

where $\Omega^{\pm}(\mathbf{k})$ denotes the Berry curvature of the effective model. In particular, by taking k_z as the free parameter in the matrix Hamiltonian defined in Eq. (4) in the main text, we have that $\Omega^{\pm}(\mathbf{k}) \equiv \Omega^{\pm}_{xy}(\mathbf{k})$, where the RHS is explicitly given by Eq. (S.22). Importantly, since k_z is treated as a parameter, we must view the Chern number as a k_z-dependent quantity. An alternative approach to calculate the Chern number consists in using the Brouwer degree of the mapping $\mathbf{k} \to \hat{\mathbf{d}}(\mathbf{k}) = \mathbf{d}(\mathbf{k})/|\mathbf{d}(\mathbf{k})|$, which leads for the following general expression (93):

$$C_{\pm} = \mp\frac{1}{2} \sum_{\mathbf{k}_D} \chi(\mathbf{k}_D)\, \mathrm{sign}[\mathrm{d_z}(\mathbf{k})] \tag{S.31}$$

where $\chi(\mathbf{k}_D) = \pm 1$ is the winding number of the Dirac point located at $\mathbf{k}_D$.

Next, to demonstrate the different topological nature of the phases in the phase diagram of Fig.1(c) of the main text, we calculate the k_z-dependent Chern number for the path in parameters' space marked with a black solid line in Fig. S2(a). The components of the $\mathbf{k}$-dependent vector $\mathbf{d}(\mathbf{k})$ for the studied case result from particularizing for $\varphi = 0$ in Eq. (5), which yields:

$$\begin{cases} d_x(\mathbf{k}) = -\sqrt{2}J\cos(k_x - \frac{\pi}{4}) - 2J\cos(k_y), \\ d_y(\mathbf{k}) = +\sqrt{2}J\cos(k_x - \frac{\pi}{4}), \\ d_z(\mathbf{k}) = -m - 2J_z\cos(k_z) + 4J'\sin(k_x)\sin(k_y), \end{cases} \tag{S.32}$$

where the corresponding Dirac points $\mathbf{k}_D$ are obtained by solving $d_x(\mathbf{k}_D) = d_y(\mathbf{k}_D) = 0$. By doing so, we found two Dirac points that we denote as:

$$\mathbf{K}_\pm = (-\frac{\pi}{4}, \pm\frac{\pi}{2}). \tag{S.33}$$

Then, in order to calculate the winding number of the Dirac points, we expand the matrix Hamiltonian around $\mathbf{K}_\pm$:

$$\bar{H}_\mathrm{B}(\mathbf{k} \sim \mathbf{K}_+) \approx (-\sqrt{2}Jq_x + 2Jq_y)\sigma_x + \sqrt{2}Jq_x\sigma_y \tag{S.34}$$

$$\bar{H}_\mathrm{B}(\mathbf{k} \sim \mathbf{K}_-) \approx (-\sqrt{2}Jq_x - 2Jq_y)\sigma_x + \sqrt{2}Jq_x\sigma_y \tag{S.35}$$

where we have defined $q_x = k_x + \pi/4$ and $q_y \mp \pi/2$. Therefore, the velocity tensors of the Dirac cones are defined as follows:

$$\bar{v}_\pm = \begin{pmatrix} -\sqrt{2}J & +\sqrt{2}J \\ \pm 2J & 0 \end{pmatrix}, \tag{S.36}$$

and the winding numbers are given by $\chi(\mathbf{K}_\pm) = \mathrm{sign}[\det(\bar{v}_\pm)]$, which yields:

$$\chi(\mathbf{K}_\pm) = \mp 1 \tag{S.37}$$

Finally, using the formula given by Eq. (S.31), we obtain:

$$\begin{aligned} C_\pm(k_z) = \mp\frac{1}{2}\Big(&\mathrm{sign}\left[-m - 2J_z\cos(k_z) + 2\sqrt{2}J'\right] - \\ &\mathrm{sign}\left[-m - 2J_z\cos(k_z) - 2\sqrt{2}J'\right]\Big) \end{aligned} \tag{S.38}$$

The calculated Chern number corresponding to the lower band $C_-(k_z)$ is plotted in Fig. S2(a) as a function of k_z and the path followed in the parameter space. As seen, $C_-(k_z) = 0$ for all k_z's when the system is in the BI phase, and $C_-(k_z) = +1$ for all k_z's when the system is in the QHI phase. When the system is in the WSM_1 phase the calculated topological invariant is not homogeneous along the considered k_z range.

3 Berry curvature of the Weyl semimetal slab

One of the fundamental aspects of Weyl semimetals is the fact that Weyl points behave as monopoles of the Berry curvature. A naturally arising question is how this unique condition translates into the geometrical properties of the Hilbert space describing the surface states of an open Weyl environment. It turns out that the surface Berry curvature (i.e., the Berry curvature calculated over the surface Brillouin zone) of this class of topological reservoirs presents some interesting features. For example, the emergence of "hot lines" along which the Berry curvature exhibits a divergent behaviour has been demonstrated, very recently, using a continuous model (54). Interestingly, these "hot lines" are predicted to strongly affect the nonlinear Hall effect in electronic systems.

For completeness, here, we describe how we computed the Berry curvature of the bands supporting the Fermi arcs in the studied lattice scheme. We first note that the number of Bloch bands resulting from the diagonalization of H_{B} when the slab geometry is implemented coincides with the number of non-equivalent sites composing the extended unit cell. If the latter comprises an odd number of sites, the dispersion relation of the slab model will consist of two separated sets of bulk bands connected by a single edge band [see red surface in the inset of Fig. 1(d) of the main text]. For the considered slab, the $k_{\parallel}$ and k_z quasi-momentum components

are well defined. Therefore, the surface Berry curvature can be calculated as follows (62):

$$\Omega^n(\mathbf{k}) = \frac{\partial \mathcal{A}_z^n(\mathbf{k})}{\partial k_{||}} - \frac{\partial \mathcal{A}_{||}^n(\mathbf{k})}{\partial k_z} \tag{S.39}$$

where $\boldsymbol{\mathcal{A}}^n(\mathbf{k})$ is the Berry connection of the n-th band [see also Eq. (S.14) in Section 1 of the Supplementary Materials].

The color maps in Fig. 1(e-g) of the main text display the Berry curvature corresponding to the edge band $\Omega_{\text{eb}}(\mathbf{k})$, for the three different configurations marked in the phase diagram of Fig. 1(d) of the main text. The Berry curvature in the first case is always zero due to the sublattice-dependent structure of the Bloch states associated with the edge band in the slab model, but the distribution observed for the (II) and (III) configurations shows some regions for which the Berry curvature present a non-trivial value. These regions in the surface Brillouin zone are associated with areas in which the localization of the wavefunction changes drastically (54). The obtained results represent a paradigmatic example of the unique Berry-curvature effects that are predicted to appear in the surface Brillouin zone of Weyl semimetals. In particular, we show that the "hot lines" of divergent Berry curvature studied in Ref. (54) emerge also naturally in a discrete lattice model.

4 Mapping between real and reciprocal space

In this section, we formalize the mapping established between the real space propagation of the photonic excitation through the lattice sites and the Fermi arc representations illustrated in Figs. 2(b-d) of the main text. To do that, we study the photonic component of the overall-wavefunction ansatz given by Eq. (6), which we identify as:

$$|\Psi_{\text{ph}}(t)\rangle = \sum_{\mathbf{r}} C_{\mathbf{r}}(t)\, a_{\mathbf{r}}^{\dagger}\, |\text{vac}\rangle, \tag{S.40}$$

where $|\text{vac}\rangle$ denotes the electromagnetic vacuum. More precisely, we aim to rewrite $|\Psi_{\text{ph}}(t)\rangle$

as follows:

$$|\Psi_{\rm ph}(t)\rangle = \sum_{\mathbf{k}} C_{n\mathbf{k}}(t)\, \tilde{a}^{\dagger}_{n\mathbf{k}}\, |{\rm vac}\rangle, \tag{S.41}$$

where our main goal is to determine the form of the $C_{n\mathbf{k}}(t)$ coefficients. These can be physically interpreted as the projections of the overall system state at instant t over the Bloch modes that diagonalize $H_{\rm B}$, therefore, $|C_{n\mathbf{k}}(t)|^2$ represents the population of Bloch modes associated to the band n and quasi-momentum $\mathbf{k}$. We start by introducing an additional label to specifically account for the sublattice degree of freedom in Eq. (S.40):

$$|\Psi_{\rm ph}(t)\rangle = \sum_{\xi_n} \sum_{\mathbf{r}\in\xi_n} C_{\mathbf{r}}(t)\, a^{\dagger}_{\mathbf{r}}\, |{\rm vac}\rangle, \tag{S.42}$$

which, utilizing the Fourier transformation of the bosonic operators defined in Eq. (S.7), can be rewritten as:

$$|\Psi_{\rm ph}(t)\rangle = \sum_{\mathbf{k}} A^{\dagger}_{\mathbf{k}}\, \mathbf{V}(\mathbf{k},t) |{\rm vac}\rangle, \tag{S.43}$$

where $A^{\dagger}_{\mathbf{k}}$ stands for the row vector operator introduced in Eq. (S.8) and we have defined the column vector $\mathbf{V}(\mathbf{k},t)$, whose n-th component reads:

$$[\mathbf{V}(\mathbf{k},t)]_n = \frac{1}{\sqrt{N_{\xi_n}}} \sum_{\mathbf{r}\in\xi_n} C_{\mathbf{r}}(t) e^{-i\mathbf{k}\mathbf{r}} \tag{S.44}$$

Finally, we introduce the unitary transformation $\bar{U}(\mathbf{k})$ that diagonalizes the Bloch Hamiltonian $\bar{H}_{\rm B}(\mathbf{k})$ [see Eq. (S.10)], to express $|\Psi_{\rm ph}(t)\rangle$ as:

$$|\Psi_{\rm ph}(t)\rangle = \sum_{\mathbf{k}} \underbrace{A^{\dagger}_{\mathbf{k}} \bar{U}(\mathbf{k})}_{\equiv \tilde{A}^{\dagger}_{\mathbf{k}}}\, \underbrace{\bar{U}^{\dagger}(\mathbf{k}) \mathbf{V}(\mathbf{k},t)}_{\equiv \tilde{\mathbf{V}}(\mathbf{k},t)} |{\rm vac}\rangle, \tag{S.45}$$

which yields:

$$|\Psi_{\rm ph}(t)\rangle = \sum_{\mathbf{k}} [\tilde{\mathbf{V}}(\mathbf{k},t)]_n\, \tilde{a}^{\dagger}_{n\mathbf{k}} |{\rm vac}\rangle, \tag{S.46}$$

where we identify $C_{n\mathbf{k}}(t) = [\tilde{\mathbf{V}}(\mathbf{k},t)]_n$.

We would like to remark that the presented mapping procedure explicitly accounts for the sublattice structure of the bath. Furthermore, we claim that a similar strategy can be employed even in the case in which a more involved structure of the photonic environment is assumed, e.g., if additional "internal" degrees of freedom, such as orbital or spin degrees of freedom, are included.

5 Time of flight

In standard time of flight experiments, a Bose-Einstein condensate trapped into an optical potential is released resulting in the free expansion of the atomic cloud. The spatial density distribution of the ensemble is obtained via absorption imaging. In particular, after a certain expansion time, t_{ToF}, the atoms are illuminated by a resonant laser beam. Part of the injected light is absorbed by the atomic cloud casting a shadow that is recorded in a CCD camera aligned with the laser direction. The ratio between the recorded intensity pattern, $I(x, y)$, and the profile obtained when the atomic cloud is absent, $I_0(x, y)$, is related to the column density, $\tilde{n}(x, y)$, which corresponds to the total density distribution, $\tilde{n}(\mathbf{r})$, integrated along the imaging direction. The total density distribution can be calculated as follows (94):

$$\tilde{n}(\mathbf{r}) = \left(\frac{m}{\hbar t_{ToF}}\right)^3 \left|w\left(\mathbf{k} = \frac{m\mathbf{r}}{\hbar t_{ToF}}\right)\right|^2 n\left(\mathbf{k} = \frac{m\mathbf{r}}{\hbar t_{ToF}}\right), \tag{S.47}$$

where m is the mass of the atoms in the ensemble and $w(\mathbf{k})$ is the Fourier transform of the on-site Warnnier function. Following (95), we will ignore the latter function by formally setting it to unity. Then, we find that, when $\mathbf{k}$ is restricted to the first Brillouin zone, the total density distribution is essentially proportional to the quasimomentum distribution, $n(\mathbf{k})$, which reads as:

$$n(\mathbf{k}) = \sum_{j,j'} e^{i\mathbf{k}(\mathbf{r}_j - \mathbf{r}_{j'})} \langle\Psi(t)|a^\dagger_{\mathbf{r}_j} a_{\mathbf{r}_{j'}}|\Psi(t)\rangle, \tag{S.48}$$

where $|\Psi(t)\rangle$ corresponds to the overall-system wavefunction at the released time.

6 Entangling capability of the Fermi arcs light-matter interface in non-ideal configurations

In the main text, the entangling capability of the Fermi arcs light-matter interface is studied for an optimal configuration featuring minimally dispersive Fermi arcs. In this section, we analyze the entangling properties displayed by non-ideal Fermi arc interfaces and show that their entangling capability is not completely destroyed by the presence of more dispersive Fermi arcs. This is due to the unique nature of the negative refraction displayed by the Weyl systems which yields a strong focalization of the photonic beam with, potentially, no back-scattering.

To illustrate that, let us distinguish between the two regimes presented in the last part of the paper. To characterize the impact of non-ideality in the large size limit ($\tau \ll T_R$), we plot in Fig. S3 the population dynamics and concurrence of two emitters placed at adjacent facets as in Fig. 5(d) of the main text, but for a non-optimal situation corresponding to preparing the bath in the configurations II and III on the phase diagram of Fig. 1(d) in the main manuscript. There, we observe how the chiral surface modes still preserve to a large extent its entangling capability.

In the small system's limit ($\tau \gg T_R$), the situation is even more favorable. The geometry of the emerging cavity enhances the state transfer properties and, consequently, the concurrence displayed by the considered emitters features values very close to unity even if no fine-tuning of the Weyl environment is performed. In Fig. S4 we show the oscillatory dynamics and concurrence of two emitters embedded in the Weyl cavity in a situation analogous to the one presented in Fig. 6 of the main text but for the non-ideal configurations proposed above. As seen, the global behavior is similar to the one described in the main manuscript. For the configuration II, more pronounced short-time-scale oscillations than in the ideal case are shown, indicating that the τ/T_R ratio is smaller here than in the situation presented in the main text. For the configura-

tion III, incomplete emitter-emitter exchanges are observed. These are related to the presence of a staggered mass term that induces an effective detuning between the considered emitters as explained in Ref. (30), and nothing related to the dispersive nature of the wavepackets.

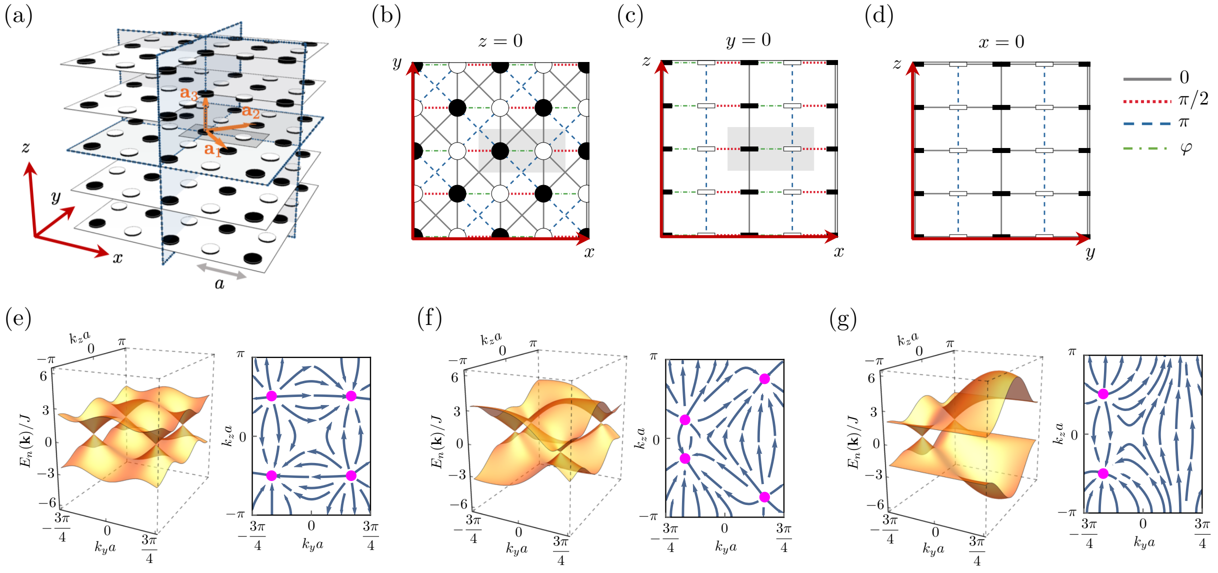

Figure S1: **Tight-binding model used to describe the photonic Weyl environment.** (a) Three-dimensional scheme of the employed lattice model. (b-d) Top and side views of the lattice model including a sketch of the employed hopping pattern. (e-g) Dispersion relation for fixed $k_x a = -\pi/4$ for the three configurations (I), (II), and (III) highlighted in the phase diagram of Fig. 1(c). Insets show the Berry curvature associated with the corresponding cuts for the lower band $\mathbf{\Omega}^{-}(\mathbf{k})$. The magenta dots denote the positions of the Weyl points.

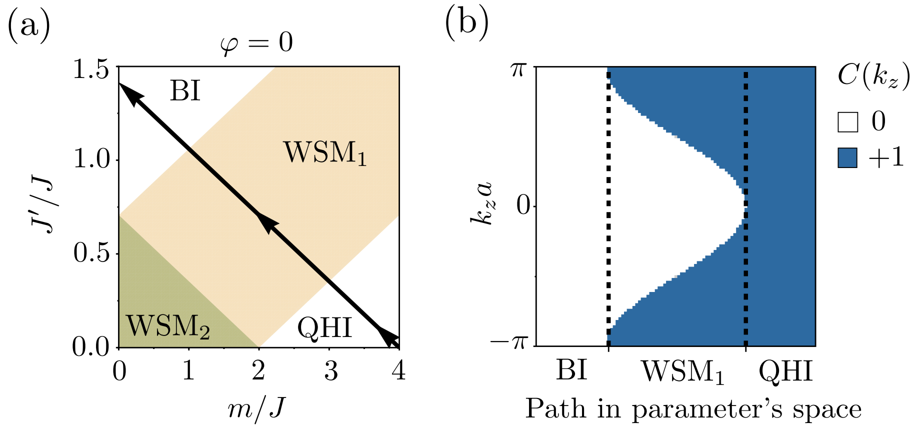

Figure S2: **Topological characterization of the gapped phases in the phase diagram.** (a) Selected path in parameters' space to demonstrate the different topological nature of the band, and quantum Hall (anomalous) insulating phases (BI and QHI, respectively). (b) Chern number calculated for the set of parameters defined by the path highlighted in panel (a).

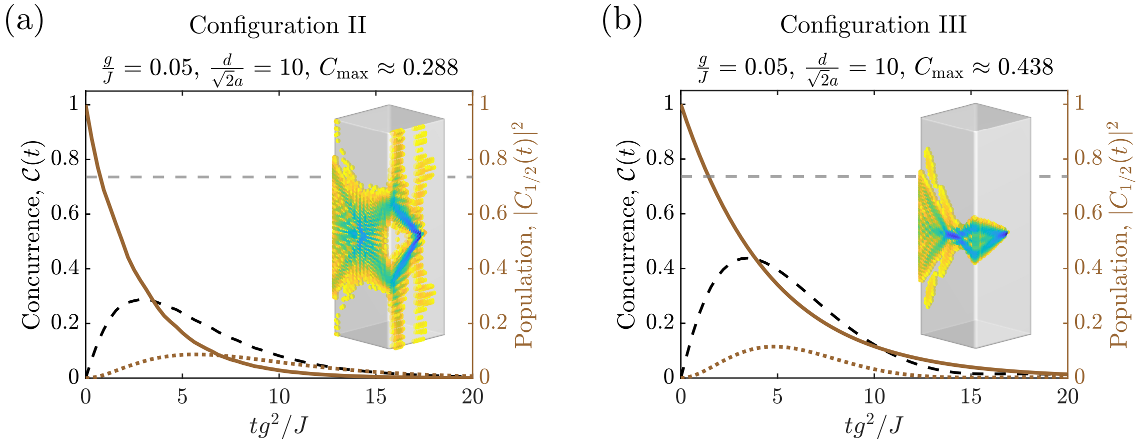

Figure S3: **Transient entanglement generation for a non-ideal Fermi arc light-matter interface.** (a) Population of the initially excited and the initially de-excited emitters (brown solid and dotted lines, respectively) and associated concurrence (dashed black) when the emitters are coupled in adjacent facets of the lattice with the Weyl system prepared in the configuration denoted by II in the phase diagram of Fig. 1(d) in the main text. The horizontal, dotted, gray line marks the maximum transient entanglement value that can be achieved for a perfect Markovian chiral quantum optical channel which is $2/e \approx 0.736$. (b) The same that in (a) but for the configuration denoted by III in the phase diagram of Fig. 1(d) in the main text. The emitters are detuned to the Weyl frequency. In both cases, we assume $g/J = 0.05$ and $d/a = 10\sqrt{2}$.

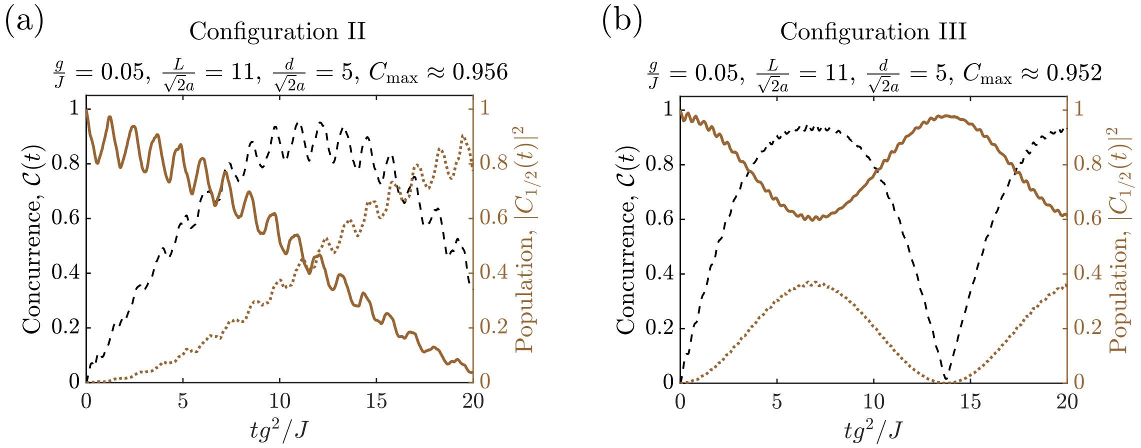

Figure S4: **Effective Fermi-arcs cavity in the non-ideal case.** (a) Population dynamics (solid and dotted brown lines) and concurrence (dashed black line) of two emitters coupled to adjacent facets of the Weyl system provided that the small system's size is considered and that the Weyl bath is prepared in the configuration denoted by II in the phase diagram of Fig. 1(d) in the main text. (b) The same that in (a) but for the configuration denoted by III in the phase diagram of Fig. 1(d) in the main text. The emitters are detuned to the Weyl frequency. In both cases, we assume $L/a = 11\sqrt{2}$, $g/J = 0.05$, and $d/a = 10\sqrt{2}$.
